# Comparative genomics reveals evolutionary loss of epiplakin in cetaceans

**DOI:** 10.1038/s41598-022-05087-0

**Published:** 2022-01-21

**Authors:** Peter Fuchs, Corinne Drexler, Sonia Ratajczyk, Leopold Eckhart

**Affiliations:** 1 Max Perutz Labs, Department of Biochemistry and Cell Biology, University of Vienna, Vienna Biocenter Campus (VBC), Vienna, Austria; 2 Skin Biology Laboratory, Department of Dermatology, Medical University of Vienna, Vienna, Austria

**Keywords:** Cytoskeleton, Evolutionary biology

## Abstract

The adaptation of vertebrates to different environments was associated with changes in the molecular composition and regulation of epithelia. Whales and dolphins, together forming the clade cetaceans, have lost multiple epithelial keratins during or after their evolutionary transition from life on land to life in water. It is unknown whether the changes in keratins were accompanied by gain or loss of cytoskeletal adapter proteins of the plakin family. Here we investigated whether plakin proteins are conserved in cetaceans and other vertebrates. Comparative analysis of genome sequences showed conservation of dystonin, microtubule actin crosslinking factor 1 (MACF1), plectin, desmoplakin, periplakin and envoplakin in cetaceans. By contrast, *EPPK1* (epiplakin) was disrupted by inactivating mutations in all cetaceans investigated. Orthologs of *EPPK1* are present in bony and cartilaginous fishes and tetrapods, indicating an evolutionary origin of *EPPK1* in a common ancestor of jawed vertebrates (Gnathostomes). In many vertebrates, *EPPK1* is flanked by an as-yet uncharacterized gene that encodes protein domains homologous to the carboxy-terminal segment of MACF1. We conclude that epiplakin, unlike other plakins, was lost in cetaceans.

## Introduction

Epithelia line the surface of the skin and cavities in internal organs. The epithelium of the skin, the epidermis, forms the interface between the organism and its environment^1,2^. Changes in epidermal structure and composition represent key steps in the evolution of vertebrates, including the emergence of an efficient protection against water loss in tetrapods and evolution of specialized skin appendages, such as scales, claws, feathers and hair^3–5^. The evolution of different lifestyles and diets has also led to modifications of internal epithelia of the lung, stomach, intestine and other organs^6,7^.

One of the key features of epithelial evolution was the adaptation of the cytoskeleton. Microfilaments and microtubules have been conserved with little or no variation whereas intermediate filaments have evolved specific compositions in many epithelial cell types with dynamic responses to stress^6,8,9^. Keratins are the characteristic intermediate filament proteins of epithelia. More than 50 keratins exist in humans with different heterodimers of type I and type II keratins being expressed in the epidermis, hair, skin glands, and stratified and simple epithelia of internal organs^6,8,9^. The diverse clades of vertebrates differ with regard to their sets of keratins. Recently, cetaceans (whales and dolphins) were shown to have a strongly reduced number of keratins, reflecting adaptations of the skin and perhaps other organs during their evolutionary transition from terrestrial to fully aquatic life^10^. Importantly, the quantitatively predominant proteins of the suprabasal epidermis of terrestrial mammals, i.e. keratins K1, K2 and K10, are absent in cetaceans because of inactivating mutations in their genes^10^.

The function of the epithelial cytoskeleton depends also on large cytoskeletal linker proteins of the plakin family, which connect microfilaments, microtubules and intermediate filaments and thereby provide stress resistance and mechanical resilience^11,12^. Seven plakin proteins, not considering isoforms produced by alternative splicing, have so far been described in mammals: dystonin (DST)/bullous pemphigoid antigen 1 (BPAG1), desmoplakin (DSP), envoplakin (EVPL), epiplakin 1 (EPPK1), microtubule actin cross-linking factor 1 (MACF1), periplakin (PPL), and plectin (PLEC)^11,13–15^. Plakins consist of multiple domains, including a plakin domain, plakin repeat domains, a growth-arrest-specific protein 2 (GAS2) domain, a calponin homology (CH) domain, EF hand, a coiled-coil rod and a spectrin repeat rod^12,13,16–18^. However, these domains are not conserved in all members of the protein family. The signifying plakin domain, consisting of a Src-homology-3 (SH3) domain flanked by spectrin repeats, is present in all plakin proteins with the exception of epiplakin^19,20^. The latter has a unique structure as it contains multiple plakin repeat domains, which mediate binding to intermediate filaments, but no other domains^21–25^. At the gene level, *EPPK1* is also unique because the entire coding sequence is present in a single exon^23,26^ and consequently, alternative splicing that generates many isoforms of other members of this gene family, does not occur in *EPPK1*.

The aim of the present study was to determine whether evolutionary adaptions of the cytoskeleton in the epidermis and other epithelia were associated with changes in plakin genes. We show that epiplakin has undergone pseudogenization in cetaceans and, upon comparative genomics analysis of vertebrates, identify a previously uncharacterized plakin gene which is located besides *EPPK1* in diverse species of fish and tetrapods and has been lost in a common ancestor of placental mammals.

## Results

### *EPPK1* is inactivated by mutations in cetaceans

**Figure 1:**
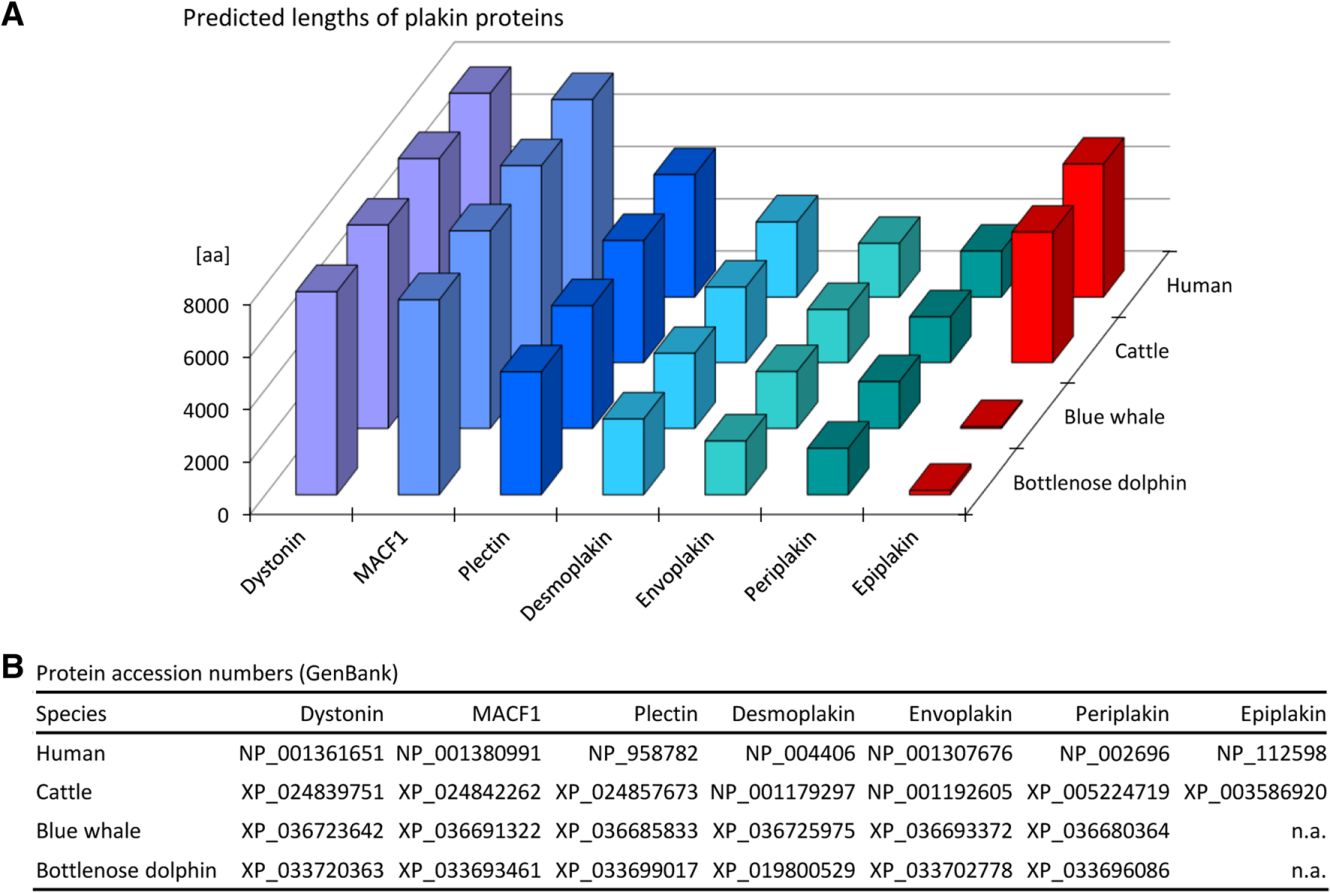
Size of plakin family proteins of land-dwelling mammals and cetaceans. (**A**) The predicted lengths of plakin family proteins are shown for human, cattle, blue whale and bottlenose dolphin. *aa* amino acid residues. (**B**) GenBank accession numbers of the proteins that were analyzed in panel (**A**). For EPPK1 of the blue whale and the bottlenose dolphin, no prediction was available in GenBank. These proteins were predicted in this study by translating the region from the conserved start codon to the first in-frame stop codon, as shown in Supplementary Table S1. *n.a.* not applicable.

We investigated the published genome sequences of cetaceans for the presence of homologs of the seven human genes of the plakin family. Genes encoding MACF1, dystonin (DST)/BPAG1, plectin, desmoplakin, envoplakin and periplakin were conserved in the blue whale (*Balaenoptera musculus*), a representative of baleen whales (Mysticeti), and in the bottlenose dolphin, a representative of toothed whales (Odontoceti), whereas only unusually short coding sequences of epiplakin could be identified in these cetaceans (Fig. 1A,B; Supplementary Table S1). The lengths of the predicted plakin proteins of cetaceans were generally similar to those of their homologs in cattle and humans (Fig. 1A). By contrast, the lengths of the theoretical EPPK1 proteins, as determined by translation of the *EPPK1* nucleotide sequence from the start codon until the first in-frame stop codon, were markedly smaller in the blue whale and bottlenose dolphin than in human and cattle (69 and 163 versus 5088 and 4997 amino acid residues, respectively) (Fig. 1A), indicating that epiplakin is not functional in cetaceans.

**Figure 2:**
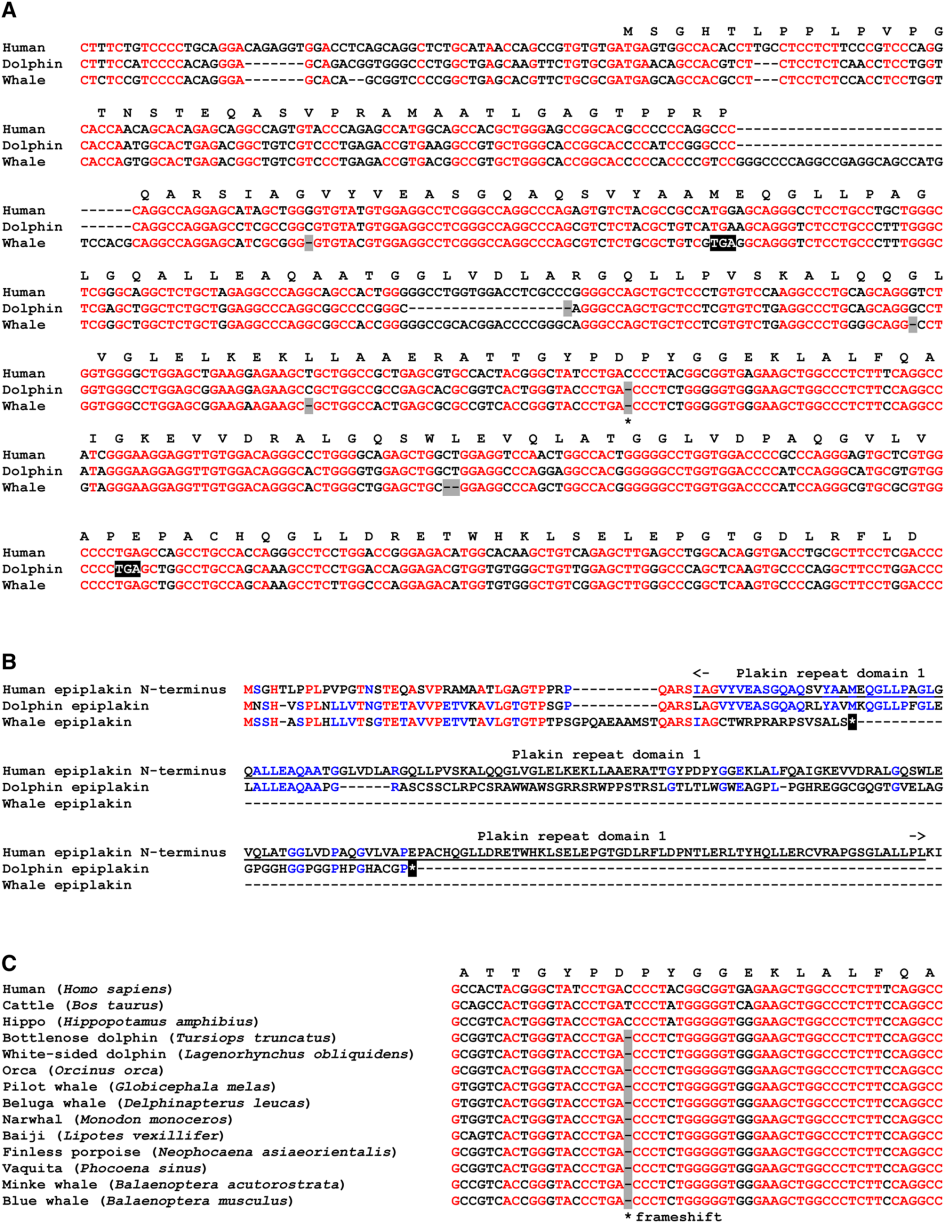
Mutations disrupt the coding sequence of *EPPK1* in cetaceans. (**A**) Alignment of partial nucleotide sequences of human, dolphin (*Tursiops truncatus*) and blue whale (*Balaenoptera musculus*) *EPPK1*. The translation of the coding sequence of human *EPPK1* is shown above the nucleotide sequences. Red fonts indicate sequence identity in all 3 species. Dashes were added to optimize the alignment. Frameshift mutations are indicated by grey shading. A black asterisk indicates a frame-shift mutation that is conserved in dolphin and whale. (**B**) Alignment of amino acid sequences of the amino-terminal segment of human epiplakin (until the end of the first plakin repeat domain) and the translation products of dolphin (*Tursiops truncatus*) and blue whale (*Balaenoptera musculus*) *EPPK1*. The end of the sequence due to a stop codon is indicated by a white asterisk on black background. The plakin repeat domain 1 of human epiplakin is underlined. Red fonts indicate identity of residues in three species and blue fonts indicate conservation in two species. (**C**) Alignment of *EPPK1* nucleotide sequences flanking a frame-shift mutation that is conserved in cetaceans [marked by the black asterisk in panel (**A**)]. Nucleotide sequences of homologous segments of *EPPK1* from human, cattle, hippo and 11 species of cetaceans were aligned. Red fonts indicate sequence identity in all species. The translation of the human coding sequence is shown above the nucleotide sequences.

Alignment of nucleotide sequences revealed multiple frame-shift mutations in the 5′ region of the *EPPK1* genes of the blue whale and dolphin (Fig. 2A), and many further frame-shifts and premature stop codons are present throughout the entire *EPPK1* genes of these species. These mutations lead to truncated proteins that share only little sequence similarity with the amino-terminus of human epiplakin and contain only a small portion of the first plakin repeat domain (Fig. 2B). Similar truncations of the open reading frame of *EPPK1* are present in 10 other species of cetaceans: white-sided dolphin (*Lagenorhynchus obliquidens*), orca (*Orcinus orca*), pilot whale (*Globicephala melas*), beluga whale (*Delphinapterus leucas*), narwhal (*Monodon monoceros*), baiji (*Lipotes vexillifer*), finless porpoise (*Neophocaena asiaeorientalis*), vaquita (*Phocoena sinus*), minke whale (*Balaenoptera acutorostrata*), and sperm whale (*Physeter catodon*) (Supplementary Fig. S1).

One single-nucleotide deletion (Fig. 2C, black asterisk) was present at the same position in both blue whale and dolphin, suggesting that this inactivating mutation has been inherited from a common ancestor. Comparison of nucleotide sequences flanking this site in other cetaceans showed the presence of the frame-shift relative to the human *EPPK1* sequence in all cetaceans for which this segment of the gene could be identified in databases (Fig. 2C). As the mutation was determined by 11 independent genome sequencing projects, we can exclude that the sequence mismatch is caused by sequencing errors. In contrast to the conservation of the *EPPK1* mutation in cetaceans, no frame-shift was detected at this site in human, cattle and the next land-dwelling or amphibious relative of cetaceans, the hippopotamus (Fig. 2C). Together, these data suggest that *EPPK1* was inactivated in a common ancestor of cetaceans, that lived between 54 million years ago (estimated divergence of the lineages leading to cetaceans and hippopotamus^27^) and 33 million years ago (estimated divergence of the lineages leading to baleen whales and toothed whales^27^).

Comparative genome sequence analysis of mammals showed conservation of *EPPK1* in all terrestrial species investigated. Analysis of the amino acid sequences encoded by *EPPK1* genes revealed that the repeat structure is conserved through evolution (Supplementary Fig. S2).

In several species the coding sequence of *EPPK1*, which is between 6000 and 12,000 nucleotides long, contains one or few apparent mismatches with the inferred coding sequence (based on GenBank protein predictions). These sequence deviations may be caused by genuine mutations or by errors of sequencing or sequence assembly. For example, the protein prediction of cattle (*Bos taurus*) EPPK1 (accession number XP_003586920.3) is labelled as “low quality” in GenBank because of a single nucleotide mismatch between inferred coding sequence and the genome sequence. However, the apparent sequence deviation of *EPPK1* in *Bos taurus* is found neither in the genome sequences of the closely related species, *Bos indicus*, nor in that of hybrid cattle (*Bos indicus* × *Bos taurus*) (Supplementary Table S2). The GenBank protein prediction of bovine EPPK1 is 4997 amino acid residues long, whereas translation of the sequence in the current genome sequence assembly would yield a protein of 2993 amino acid residues. *EPPK1* of the hippopotamus could be identified only partially on a whole genome sequence contig (accession number PVJP02910133). According to the translation of this partial gene, the minimum length of the EPPK1 protein of the hippopotamus is 2290 amino acid residues (Supplementary Fig. S3). Our analysis suggests that *EPPK1* genes encode long proteins with a characteristic plakin repeat domain-rich structure in mammals other than cetaceans. By contrast, the sequence between the EPPK1 start codon and the first in-frame stop codon does not encode a single plakin repeat domain in all cetaceans investigated (Fig. 2B and Supplementary Fig. S1), indicating a loss of function of epiplakin in this phylogenetic clade.

### *EPPK1* is conserved and flanked by a previously uncharacterized plakin gene in phylogenetically diverse Gnathostomes

**Figure 3:**
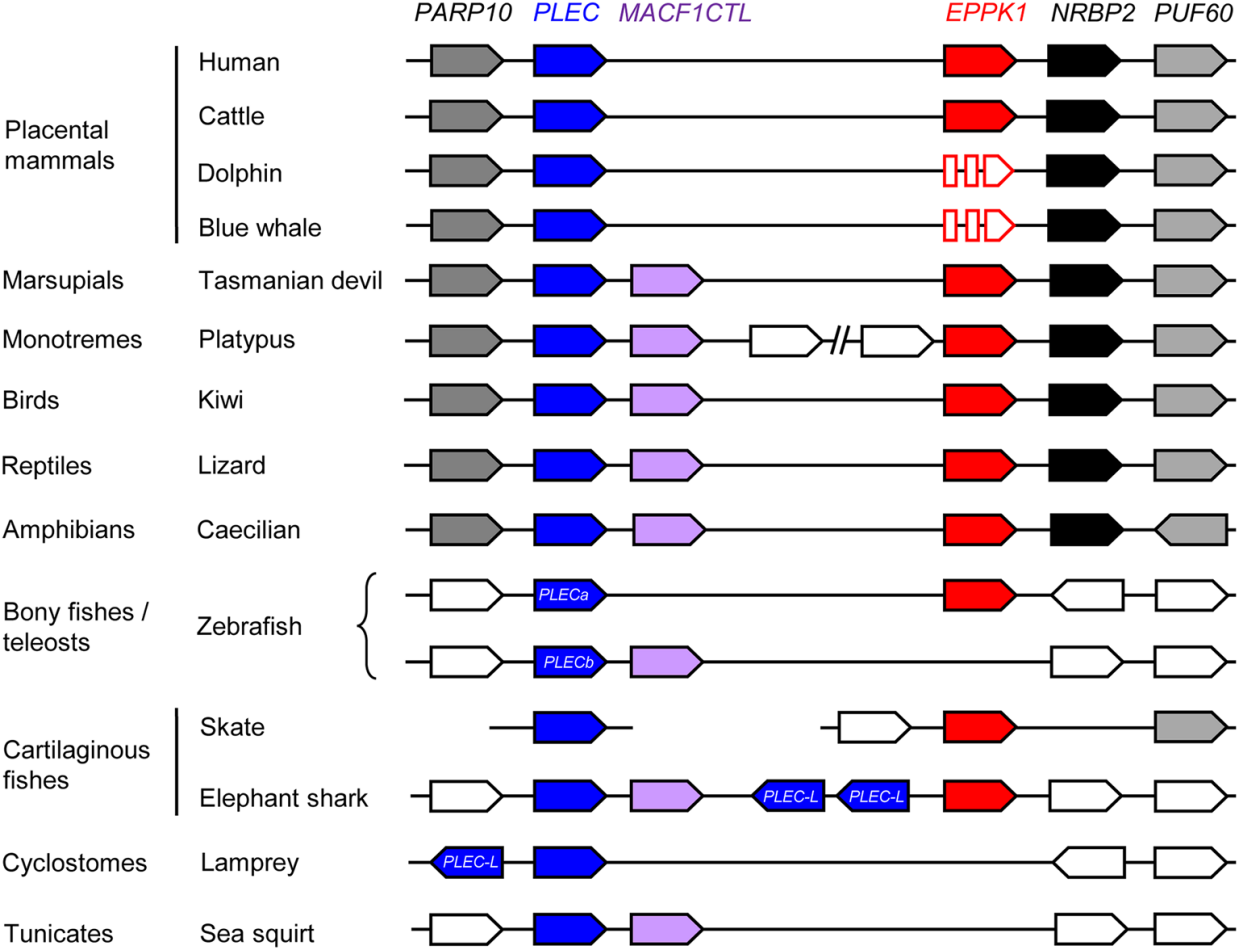
Comparison of *PLEC* and *EPPK1* gene loci among chordates. Genes are schematically depicted by arrows pointing in the direction of transcription. Colors and grey shading mark orthologous genes of different species. White arrows represent genes that are not conserved across species. Disrupted arrows indicate the presence of inactivating mutations in the *EPPK1* genes of cetaceans. The zebrafish has 2 *PLEC* genes (marked “a” and “b”) due a teleost-specific whole-genome duplication. The identity of several genes in basal chordates is uncertain. Details about the genes are provided in Supplementary Table S1. A break in the horizontal line indicates that not all genes located between *MACF1CTL* and *EPPK1* of the platypus are shown. The *PLEC* gene of the skate is located on a genomic sequence scaffold not yet placed into the current genome assembly. Note that *EPPK1* is present in jawed vertebrates (Gnathostomata) but not in lamprey (Cyclostomata) and sea squirt (Urochordata); Species: Human (*Homo sapiens*), cattle (*Bos taurus*), blue whale (*Balaenoptera musculus*), bottlenose dolphin (*Tursiops truncatus*), tasmanian devil (*Sarcophilus harrisii*), platypus (*Ornithorhynchus anatinus*), kiwi (*Apteryx rowi*), caecilian (*Rhinatrema bivittatum*), zebrafish (*Danio rerio*), skate (*Amblyraja radiata*), lamprey (*Petromyzon marinus*), sea squirt (*Ciona intestinalis*). Genes: *EPPK1*, epiplakin 1; *MACF1CTL*, microtubule actin cross-linking factor 1 carboxy-terminus-like; *PLEC*, plectin; *PLEC-L*, PLEC-like.

Next we extended our comparative analysis of *EPPK1* to non-mammalian species because *EPPK1* homologs were recently reported also for the chicken^28^, Xenopus frogs and zebrafish^29^. We investigated the gene locus of *EPPK1* in diverse vertebrates using published genome sequences available in GenBank. To this end we searched for *EPPK1* and homologs of the genes that flank *EPPK1* in the human genomes, i.e. *PARP10* and *PLEC*, which are located on the 5′-side, and *NRBP2* and *PUF60*, which are located on the 3′-side of *EPPK1* (Fig. 3). All these genes are conserved in cattle and other placental mammals (Fig. 3). *EPPK1* and neighboring genes were found in marsupials, monotremes, birds, reptiles and amphibians, suggesting that this gene arrangement and the presence of *EPPK1* represents the ancestral condition of tetrapods. However, we identified an additional gene located between *PLEC* and *EPPK1* in non-placental mammals and non-mammalian vertebrates (Fig. 3). This gene, tentatively named MACF1 carboxy-terminus-like (*MACF1CTL*), encodes a protein showing clear similarity to the carboxy-terminal segment of MACF1, including spectrin repeats, an EF hand and a GAS2 domain (Supplementary Fig. S4). Further genes are present between *MACF1CTL* and *EPPK1* in the platypus (Fig. 3), possibly indicating a clade-specific chromosomal rearrangement in monotremes.

**Figure 4:**
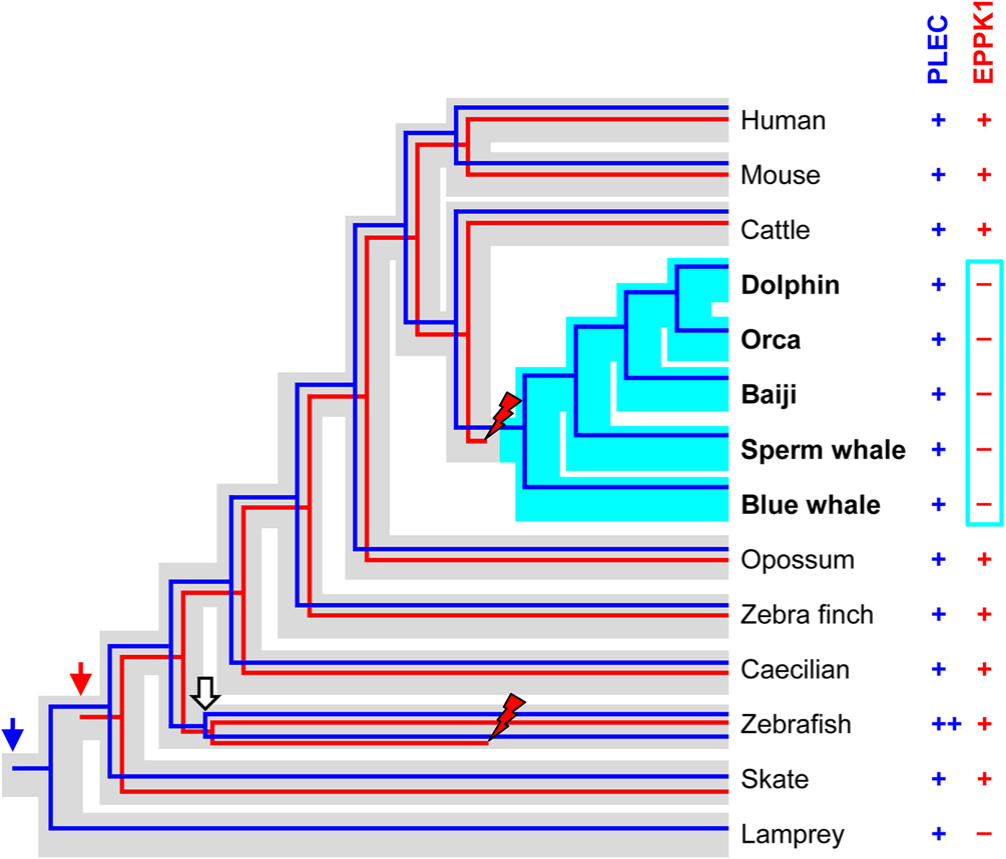
Schematic model of the origin and loss of *EPPK1* during the evolution of vertebrates. The presence (+ , 1 gene copy; ++ , 2 gene copies) or absence (–) of intact *PLEC* and *EPPK1* genes was used to infer the origin (vertical colored arrows) of *PLEC* (blue) and *EPPK1* (red) genes and the loss of the capacity of *EPPK1* to encode a functional plakin protein (red flash symbols) in cetaceans. The white arrow indicates a whole-genome duplication in teleosts. Phylogenetic trees of genes are shown within the species tree (grey for non-cetaceans, light blue for cetaceans).

In zebrafish there are two *PLEC* orthologs, resulting from gene duplication in the course of a whole genome duplication during the evolution of teleost fishes^30^. *EPPK1* is located besides one of the *PLEC* genes whereas a *MACF1CTL* gene is located besides the other *PLEC* homolog (Fig. 3), indicating that single copies of the latter genes have been conserved in zebrafish. Of note, the genes flanking *PLEC* and *EPPK1* in tetrapods are located elsewhere in the zebrafish genome. In cartilaginous fishes (Chondrichthyes), which diverged around 470 million years ago from the lineage leading to bony fishes (Osteichthyes) including tetrapods^27^, we identified an *EPPK1* gene in the genome of the thorny skate (Fig. 3). The gene is located close to *PUF60*, indicating partial conservation of the gene arrangement (synteny) relative to tetrapods. *PLEC* is located on another genome sequence scaffold, and a continuous sequence of the skate genome was not available at the time of our study. In another cartilaginous fish, the elephant shark, *PLEC* was flanked by *MACF1CTL* and by genes with similarities to plectin and epiplakin. *EPPK1* homologs could be identified in the genomes of neither the sea lamprey, a representative of basal vertebrates, nor the sea squirt (*Ciona intestinalis*) a representative of basal chordates (Fig. 3). Together, these data suggest that *EPPK1* is an evolutionarily ancient protein of Gnathostomes that has been conserved in diverse species but not in cetaceans (Fig. 4).

## Discussion

The results of the present study suggest that *EPPK1* originated more than 450 million years ago in a common ancestor of Gnathostomes and that it was inactivated by mutations in cetaceans. *EPPK1* is expressed constitutively in the epidermis and in a stress-inducible manner in various epithelia of humans and the biomedical model species mouse, but its expression patterns and functions in other animals are not known. The high degree of conservation in many lineages of Gnathostomes suggests a critical function of *EPPK1*, whereas its loss in cetaceans indicates that its function is dispensable at least under some environmental and lifestyle-associated conditions. Indeed, *EPPK1* knockout mice showed no obvious phenotype under standard laboratory housing conditions and only minor defects under stress conditions^22,31–35^, suggesting that EPPK1 is not essential for homeostatic life of mice and a possibly critical role of EPPK1 may be effective under as-yet unidentified circumstances.

The water-to-land transition of cetaceans was associated with genetic adaptations that manifest in major anatomical and physiological changes. The epidermis of whales and dolphins is extremely thick and devoid of a granular layer. Many skin barrier-related genes have been lost^28,36–38^ and the number of keratin genes is greatly reduced in cetaceans^10,39^. In addition, genes related to kidney, heart, lung, eye, ear and nervous system development have been under positive selection in cetaceans^40–42^, indicating adaptations of other organs. We hypothesize that the loss of *EPPK1* in cetaceans was facilitated by the changes of the site of highest *EPPK1* expression levels, which are the outer layers of the epidermis in other amniotes. However, *EPPK1* must have important functions unrelated to the epidermal cornification because it originated and has been conserved in aquatic animals that lack a cornified epidermis (Fig. 3). In human liver, pancreas, colon and other organs, expression of *EPPK1* is low under homeostasis^23^ but upregulated upon different types of stress^22,35,43^. The evolutionary history of *EPPK1* suggests that its primordial function is related to stress responses. It remains to be investigated which epithelial stress response mechanisms depend on *EPPK1* in most Gnathostomes but not in cetaceans. It will be interesting to find out whether the functions of *EPPK1* are not required in cetaceans because the relevant stress to skin, liver, pancreas and colon is missing or if these types of stress are counteracted by mechanisms that substitute for *EPPK1*.

Epiplakin is unique among plakin family proteins because its structure is comprised of plakin repeat domains only. Our data demonstrate that the repeat organization of EPPK1 is evolutionarily conserved (Supplementary Fig. S2), confirming the results of a recent report on EPPK1 in a selection of model species^29^. The number of repeats differs among species and it is likely that even within species copy number variations exist, as exemplified by published data on human EPPK1^44^ and mouse EPPK1^29^. Experience with human and mouse *EPPK1* gene has shown that the exact numbers of plakin repeat domains are difficult to determine by standard cloning and sequencing techniques and optimized long-range PCRs are required to ascertain the primary structure of EPPK1 in individuals of a broad range of species.

Previous data on the tandem arrangement of *PLEC* and *EPPK1* genes in the human and mouse genomes and the observation that the repeat domains of EPPK1 show the highest sequence similarity to those of plectin^17^ have suggested that *EPPK1* arose by partial tandem duplication of the *PLEC* gene. Our comparison of the *PLEC* and *EPPK1* gene loci across taxa of vertebrates reveals the existence of an as-yet uncharacterized gene, tentatively named *MACF1CTL*, located between *PLEC* and *EPPK1* in fish, amphibians, reptiles, birds and basal mammals. These data suggest that the plakin repeat domain-coding part of *PLEC*, known from humans and mouse, was originally not a direct neighbor of *EPPK1*. Accordingly, *MACF1CTL* should be taken into account in hypotheses about the evolutionary origin of *EPPK1*.

*MACF1CTL* is the eighth member of the plakin family in mammals. Of note, a ninth plakin family member may be encoded by an as-yet uncharacterized envoplakin-like (*EVPLL*) in the human genome (GenBank gene ID: 645027). Due to gene loss, *MACF1CTL* is absent in placental mammals, including both cetaceans and humans. The protein domains of plectin are homologous to the amino-terminus of MACF1, whereas the protein domains of MACF1CTL are homologous to the carboxy-terminus of MACF1 (Supplementary Fig. S4). In some species, such as the zebra finch, the gene prediction algorithm used in GenBank has yielded a single gene (GenBank gene ID: 100223531) that connects the exons of *PLEC* and *MACF1CTL*. Further investigations of actual gene expression products are necessary to determine the structure and functions of MACF1CTL. Given the homology relationships of the protein domains of MACF1, PLEC and MACF1CTL, we put forward the hypothesis that *PLEC* and *MACF1CTL* evolved by the mechanism of gene fission^45^ from a single ancestral gene that was a copy of *MACF1*. The evolutionary origin of *EPPK1* may have coincided with this gene recombination event.

In conclusion, our comparative genomics data suggest that *EPPK1* is an evolutionarily ancient gene of jawed vertebrates, which has been lost during the water-to-land transition of cetaceans. As *EPPK1* is expressed at particularly high levels in the epidermis of terrestrial mammals, the loss of function of epiplakin may contribute to unique features of the epidermal cytoskeleton in cetaceans.

## Methods

### Ethics statement

Genes were investigated exclusively using sequences available in public databases. This research did not include human or animal subjects.

### Identification and comparative analysis of plakin family genes

Orthology of genes was determined using the criteria of gene locus synteny and best reciprocal Basic Local Alignment Search Tool (BLAST) hits of the encoded protein sequences. Nucleotide sequence were translated into amino acid sequences using the Translate tool at the Expasy website of the Swiss Institute of Bioinformatics (https://web.expasy.org/translate/). The domain structure of proteins was analyzed with the NCBI conserved domains tool (https://www.ncbi.nlm.nih.gov/Structure/cdd/wrpsb.cgi). Nucleotide and amino acid sequence were aligned with MUSCLE (https://www.ebi.ac.uk/Tools/msa/muscle/). Amino acid sequence repeats in EPPK1 proteins were identified by dot plot analysis in which all parts of the protein were compared with all other parts of the same protein. Dot plots were generated by Dotmatcher (https://www.bioinformatics.nl/cgi-bin/emboss/dotmatcher) using default settings of “Window size over which to test threshold”: 10 and “Threshold”: 23, whereby input sequence 1 and input sequence 2 were identical. Origin and loss of genes during evolution were inferred from the pattern of presence or absence of orthologous genes in extant species according to the criterion of maximum parsimony.

## Supplementary Information

Supplementary Information.

## Data Availability

All data generated or analyzed during this study are included in this published article and its Supplementary Information files.
