# Supplementary material for: Comparative genomics reveals evolutionary loss of epiplakin in cetaceans
Source: Sci Rep. 2022 Jan 21;12:1112. doi: 10.1038/s41598-022-05087-0 (PMC8782857; doi:10.1038/s41598-022-05087-0)
Supplement: Supplementary file 1 — Supplementary Information. [file 41598_2022_5087_MOESM1_ESM.pdf]

Supplementary Data: Supplementary Tables and Figures

Comparative genomics reveals evolutionary loss of epiplakin in cetaceans

Peter Fuchs, Corinne Drexler, Sonia Ratajczyk, Leopold Eckhart

Content

Supplementary Tables S1-S2

Supplementary Figures S1-S4

Supplementary Table S1. Genes and nucleotide sequences investigated in this study

Species	Species	Gene	GenBank gene ID	Notes	Results of analysis				
Human	*Homo sapiens*	*DST*	667		Figure 1				
Cattle	*Bos taurus*	*DST*	535297		Figure 1				
Blue whale	*Balaenoptera musculus*	*DST*	118903071		Figure 1				
Bottlenose dolphin	*Tursiops truncatus*	*DST*	101331330		Figure 1				
Human	*Homo sapiens*	*MACF1*	23499		Figure 1				
Cattle	*Bos taurus*	*MACF1*	506730		Figure 1				
Blue whale	*Balaenoptera musculus*	*MACF1*	118886151		Figure 1				
Bottlenose dolphin	*Tursiops truncatus*	*MACF1*	101326574		Figure 1				
Human	*Homo sapiens*	*PLEC*	5339		Figure 1		Figure 3	Figure 4	
Cattle	*Bos taurus*	*PLEC*	786966		Figure 1		Figure 3	Figure 4	
Blue whale	*Balaenoptera musculus*	*PLEC*	118883296		Figure 1		Figure 3	Figure 4	
Bottlenose dolphin	*Tursiops truncatus*	*PLEC*	101318066		Figure 1		Figure 3	Figure 4	
Human	*Homo sapiens*	*DSP*	1832		Figure 1				
Cattle	*Bos taurus*	*DSP*	514360		Figure 1				
Blue whale	*Balaenoptera musculus*	*DSP*	118904192		Figure 1				
Bottlenose dolphin	*Tursiops truncatus*	*DSP*	101335328		Figure 1				
Human	*Homo sapiens*	*EVPL*	2125		Figure 1				
Cattle	*Bos taurus*	*EVPL*	510502		Figure 1				
Blue whale	*Balaenoptera musculus*	*EVPL*	118887001		Figure 1				
Bottlenose dolphin	*Tursiops truncatus*	*EVPL*	117309406		Figure 1				
Human	*Homo sapiens*	*PPL*	5493		Figure 1				
Cattle	*Bos taurus*	*PPL*	522886		Figure 1				
Blue whale	*Balaenoptera musculus*	*PPL*	118880683		Figure 1				
Bottlenose dolphin	*Tursiops truncatus*	*PPL*	101327255		Figure 1				
Human	*Homo sapiens*	*EPPK1*	83481		Figure 1	Figure 2	Figure 3	Figure 4	
Cattle	*Bos taurus*	*EPPK1*	100337278		Figure 1	Figure 2	Figure 3	Figure 4	
Blue whale	*Balaenoptera musculus*	*EPPK1*	118883774	NC_045801.1, nucl. 793885-794094 (start - stop codon)	Figure 1	Figure 2		Figure 4	
Bottlenose dolphin	*Tursiops truncatus*	*EPPK1*	n.a.	NC_047050.1, nucl. 77575431-77574940 (start - stop codon)	Figure 1	Figure 2		Figure 4	
White-sided dolphin	*Lagenorhynchus obliquidens*	*EPPK1*	n.a.	NW_020837985.11, sequence data in Suppl. Fig. S1		Figure 2			Suppl. Fig. S1
Orca	*Orcinus orca*	*EPPK1*	n.a.	NW_004438426.1, sequence data in Suppl. Fig. S1		Figure 2		Figure 4	Suppl. Fig. S1
Pilot whale	*Globicephala melas*	*EPPK1*	n.a.	SWEB01005905.1, sequence data in Suppl. Fig. S1		Figure 2			Suppl. Fig. S1
Beluga whale	*Delphinapterus leucas*	*EPPK1*	n.a.	NW_022098049.1, sequence data in Suppl. Fig. S1		Figure 2			Suppl. Fig. S1
Narwhal	*Monodon monoceros*	*EPPK1*	n.a.	RWIC01000405.1, sequence data in Suppl. Fig. S1		Figure 2			Suppl. Fig. S1
Baiji	*Lipotes vexillifer*	*EPPK1*	n.a.	AUPI01111971.1, sequence data in Suppl. Fig. S1		Figure 2		Figure 4	Suppl. Fig. S1
Finless porpoise	*Neophocaena asiaeorientalis*	*EPPK1*	n.a.	NW_020172779.1, sequence data in Suppl. Fig. S1		Figure 2			Suppl. Fig. S1
Vaquita	*Phocoena sinus*	*EPPK1*	n.a.	NC_045779.1, sequence data in Suppl. Fig. S1		Figure 2			Suppl. Fig. S1
Minke whale	*Balaenoptera acutorostrata*	*EPPK1*	n.a.	SWEB01005905.1, sequence data in Suppl. Fig. S1		Figure 2			Suppl. Fig. S1
Sperm whale	*Physeter catodon*	*EPPK1*	102989592	NW_021146471.1, sequence data in Suppl. Fig. S1				Figure 4	Suppl. Fig. S1
Hippopotamus	*Hippopotamus amphibius*	*EPPK1*	n.a.						Suppl. Fig. S3
Tasmanian devil	*Sarcophilus harrisii*	*EPPK1*	100920438				Figure 3		
Tasmanian devil	*Sarcophilus harrisii*	*MACF1*	100915764						Suppl. Fig. S4
Tasmanian devil	*Sarcophilus harrisii*	*MACF1CTL*	111719160	LOC111719160, microtubule-actin cross-linking factor 1-like			Figure 3		Suppl. Fig. S4
Tasmanian devil	*Sarcophilus harrisii*	*PLEC*	100920957				Figure 3		Suppl. Fig. S4
Platypus	*Ornithorhynchus anatinus*	*PLEC*	100076841				Figure 3		
Platypus	*Ornithorhynchus anatinus*	*MACF1CTL*	103165878	LOC103165878, microtubule-actin cross-linking factor 1-like			Figure 3		
Platypus	*Ornithorhynchus anatinus*	*EPPK1*	103168963				Figure 3		
Kiwi	*Apteryx rowi*	*PLEC*	112976771				Figure 3		
Kiwi	*Apteryx rowi*	*MACF1CTL*	112976772	LOC112976772, microtubule-actin cross-linking factor 1-like			Figure 3		
Kiwi	*Apteryx rowi*	*EPPK1*	112976765	GenBank prediction needs corrections			Figure 3		
Caecilian	*Rhinatrema bivittatum*	*EPPK1*	115085809				Figure 3	Figure 4	
Zebrafish	*Danio rerio*	*EPPK1*	101885652				Figure 3	Figure 4	
Skate	*Amblyraja radiata*	*PLEC*	116969968				Figure 3		
Skate	*Amblyraja radiata*	*EPPK1*	116985715				Figure 3	Figure 4	
Elephant shark	*Callorhinchus milii*	*PLEC*	103179737				Figure 3		
Elephant shark	*Callorhinchus milii*	*MACF1CTL*	103179735	LOC103179735, microtubule-actin cross-linking factor 1-like			Figure 3		
Elephant shark	*Callorhinchus milii*	*EPPK1-like*	103179662				Figure 3		
Lamprey	*Petromyzon marinus*	*PLEC*	116945257				Figure 3	Figure 4	
Lamprey	*Petromyzon marinus*	*MACF1*	116941622						Suppl. Fig. S4
Sea squirt	*Ciona intestinalis*	*PLEC*	113474048	gene identity uncertain			Figure 3	Figure 4	
Sea squirt	*Ciona intestinalis*	*MACF1CTL*	100175356	gene identity uncertain			Figure 3		
Sperm whale	*Physeter catodon*	*PLEC*	102989860					Figure 4	
Baiji	*Lipotes vexillifer*	*PLEC*	103076392					Figure 4	
Orca	*Orcinus orca*	*PLEC*	101271232					Figure 4	
Opossum	*Monodelphis domestica*	*EPPK1*	100017139					Figure 4	
Zebra finch	*Taeniopygia guttata*	*EPPK1*	115492384					Figure 4	

This table includes information about the genes of main interest in the figures and text of the manuscript. Other genes can be identified in GenBank using the positional information provided in Figure 3.
Note that the identity and exon structure of several genes is uncertain. This uncertainty affects particularly species, that are phylogenetically far away from mammals.

Supplementary Table S2. Comparative analysis of a nucleotide sequence mismatch in one repeat of bovine EPPK1

Species/breed	Acc. nr.	Start	Nucleotide sequence	End
Bos taurus	NC_037341.1	947988	CGCGACACCCGCGTGACGCTGGGCGCCGGCCCCCCGCGGGGCCAGCGCGTCTCCGTGTGG	948047
Bos taurus	NC_037341.1	949536	CGCGACACCCGCGTGACGCTGGGCGCCGG-CCCCCGCGGGGCCAGCGCGTCTCCGTGTGG	949594
Bos taurus	NC_037341.1	951083	CGCGACACCCGCGTGACGCTGGGCGCCGGCCCCCCGCGGGGCCAGCGCGTCTCCGTGTGG	951142
Bos taurus	NC_037341.1	952631	CGCGACACCCGCGTGACGCTGGGCGCCGGCCCCCCGCGGGGCCAGCGCGTCTCCGTGTGG	952690
Bos taurus	NC_037341.1	954179	CGCGACACCCGCGTGACGCTGGGCGCCGGCCCCCCGCGGGGCCAGCGCGTCTCCGTGTGG	954238
Bos indicus x Bos taurus	NC_040089.1	892616	CGCGACACCCGCGTGACGCTGGGCGCCGGCCCCCCGCGGGGCCAGCGCGTCTCCGTGTGG	892675
Bos indicus x Bos taurus	NC_040089.1	894164	CGCGACACCCGCGTGACGCTGGGCGCCGGCCCCCCGCGGGGCCAGCGCGTCTCCGTGTGG	894223
Bos indicus x Bos taurus	NC_040089.1	895712	CGCGACACCCGCGTGACGCTGGGCGCCGGCCCCCCGCGGGGCCAGCGCGTCTCCGTGTGG	895771
Bos indicus x Bos taurus	NC_040089.1	897260	CGCGACACCCGCGTGACGCTGGGCGCCGGCCCCCCGCGGGGCCAGCGCGTCTCCGTGTGG	897319
Bos indicus x Bos taurus	NC_040089.1	898808	CGCGACACCCGCGTGACGCTGGGCGCCGGCCCCCCGCGGGGCCAGCGCGTCTCCGTGTGG	898867
Bos indicus x Bos taurus	NC_040089.1	900356	CGCGACACCCGCGTGACGCTGGGCGCCGGCCCCCCGCGGGGCCAGCGCGTCTCCGTGTGG	900415
Bos indicus x Bos taurus	NC_040089.1	901904	CGCGACACCCGCGTGACGCTGGGCGCCGGCCCCCCGCGGGGCCAGCGCGTCTCCGTGTGG	901963
Bos indicus	NC_032663.1	765095	CGCGACACCCGCGTGACGCTGGGCGCCGGCCCCCCGCGGGGCCAGCGCGTCTCCGTGTGG	765154
Bos indicus	NC_032663.1	766643	CGCGACACCCGCGTGACGCTGGGCGCCGGCCCCCCGCGGGGCCAGCGCGTCTCCGTGTGG	766702

Nucleotide sequences of *EPPK1* gene segments of *Bos taurus* (cattle), *Bos indicus* (zebu cattle)] and *Bos indicus x Bos taurus* (hybrid cattle) were compared. Accession numbers (Acc. nr.) and start and end positions of the nucleotide sequences are indicated. A dash indicates a missing nucleotide due to a deletion or sequencing error in one repeat of *Bos taurus EPPK1*. All other sequences are 100% identical.

A

White-sided dolphin (*Lagenorhynchus obliquidens*)

```
>NW_020837985.1:c16973222-16972663 Lagenorhynchus obliquidens isolate
GAN/ISIS:26980383/991018 unplaced genomic scaffold, ASM367639v1
scaffold41, whole genome shotgun sequence
ATGAACAGCCACGTCTCTCCTCTCAACCTCCTGGTCACCAATGGCACTGAGACGGCTGTCGTCCCTGAGAC
CGTGAAGGCCGTGCTGGGCACCGGCACCCCATCTGGGCCCCAGGCCAGGAGCCTCGCCGGCGTGTATGTGG
AGGCCTCGGGCCAGGCCCAGCGTCTCTACGCTGTCATGAAGCAGGGTCTCCTGCCCTTTGGGCTCGAGCTG
GCTCTGCTGGAGGCCCAGGCGGCCCTGGGCAGGGCCAGCTGCTCCTCGTGTCTGAGGCCCTGCAGCAGGGC
CTGGTGGGCCTGGAGCGGAAGGAGAAGCCGCTGGCCGCCGAGCACGCGGTCACTGGGTACCCTGACCCTCT
GGGGGTGGGAAGCTGGCCCTCTTCCAGGCCATAGGGAAGGAGGTTGTGGACAGGGCACTGGGGTGGAGCTG
GCTGGAGGCCCAGGAGGCCACGGGGGGCCTGGTGGACCCCATCCAGGGCATGCGTGTGGCCCCTGAGCTGG
CCTGCCAGCAAAGCCTCCTGGACCAGGAGACGTGGTGTGGGCTGTTGGAGCTTGGGCCCAGCT
```

<u>Translation:</u>

```
atgaacagccacgtctctcctctcaacctcctggtcaccaatggcactgagacggctgtc
 M  N  S  H  V  S  P  L  N  L  L  V  T  N  G  T  E  T  A  V
gtccctgagaccgtgaaggccgtgctgggcaccggcaccccatctgggccccaggccagg
 V  P  E  T  V  K  A  V  L  G  T  G  T  P  S  G  P  Q  A  R
agcctcgccggcgtgtatgtggaggcctcgggccaggcccagcgtctctacgctgtcatg
 S  L  A  G  V  Y  V  E  A  S  G  Q  A  Q  R  L  Y  A  V  M
aagcagggtctcctgccctttgggctcgagctggctctgctggaggcccaggcggccctg
 K  Q  G  L  L  P  F  G  L  E  L  A  L  L  E  A  Q  A  A  L
ggcagggccagctgctcctcgtgtctgaggccctgcagcagggcctggtgggcctggagc
 G  R  A  S  C  S  S  C  L  R  P  C  S  R  A  W  W  A  W  S
ggaaggagaagccgctggccgccgagcacgcggtcactgggtaccctgaccctctggggg
 G  R  R  S  R  W  P  P  S  T  R  S  L  G  T  L  T  L  W  G
tgggaagctggccctcttccaggccatagggaaggaggttgtggacagggcactggggtg
 W  E  A  G  P  L  P  G  H  R  E  G  G  C  G  Q  G  T  G  V
gagctggctggaggcccaggaggccacggggggcctggtggaccccatccagggcatgcg
 E  L  A  G  G  P  G  G  H  G  G  P  G  G  P  H  P  G  H  A
tgtggcccctgagctggcctgccagcaaagcctcctggaccaggagacgtggtgtgggct
 C  G  P  -  A  G  L  P  A  K  P  P  G  P  G  D  V  V  W  A
gttggagcttgggcccag
 V  G  A  W  A  Q
```

B

Orca (*Orcinus orca*)

```
>NW_004438426.1:819180-819739 Orcinus orca isolate Morgan unplaced
genomic scaffold, Oorc_1.1 Scaffold12, whole genome shotgun sequence
ATGAACAGCCACGTCTCTCCTCTCAACCTCCTGGTCACCAATGGCGCTGAGACGGCTGTCGTCCCTGAGAC
CGTGAAGGCCGTGCTGGGCGCCGGCACCCCATCCGGGCCCCAGGCCAGGAGCCTCGCCGGCGCGTACGTGG
AGGCCTCGGGCCAGGCCCAGCGTCTCTACGCTGTCATGAAGCAGGGTCTCCTGCCCTTTGGGCTCGAGCTG
GCTCTGCTGGAGGCCCAGGCGGCCCCGGGCAGGGCCAGCTGCTCCTCGTGTCTGAGGCCCTGCAGCAGGGC
CTGGTGGGCCTGGAGCGGAAGGAGAAGCCGCTGGCCACCGAGCACGCGGTCACTGGGTACCCTGACCCTCT
GGGGGTGGGAAGCTGGCCCTCTTCCAGGCCATAGGGAAGGAGGTTGTGGACAGGGCACTGGGGTGGAGCTG
```

GCTGGAGGCCCAGGAGGCCACGGGGGGCCTGGTGGACCCCATCCAGGGCATGCGCGTGGCCCCTGAGCTGG
CCTGCCAGCAAAGCCTCCTGGACCAGGAGACGTGGTGTGGGCTGTTGGAGCTTGGGCCCAGCT

Translation:

```
atgaacagccacgtctctcctctcaacctcctggtcaccaatggcgctgagacggctgtc
 M  N  S  H  V  S  P  L  N  L  L  V  T  N  G  A  E  T  A  V
gtccctgagaccgtgaaggccgtgctgggcgccggcaccccatccgggccccaggccagg
 V  P  E  T  V  K  A  V  L  G  A  G  T  P  S  G  P  Q  A  R
agcctcgccggcgcgtacgtggaggcctcgggccaggcccagcgtctctacgctgtcatg
 S  L  A  G  A  Y  V  E  A  S  G  Q  A  Q  R  L  Y  A  V  M
aagcagggtctcctgccctttgggctcgagctggctctgctggaggcccaggcggccccg
 K  Q  G  L  L  P  F  G  L  E  L  A  L  L  E  A  Q  A  A  P
ggcagggccagctgctcctcgtgtctgaggccctgcagcagggcctggtgggcctggagc
 G  R  A  S  C  S  S  C  L  R  P  C  S  R  A  W  W  A  W  S
ggaaggagaagccgctggccaccgagcacgcggtcactgggtaccctgaccctctgggg
 G  R  R  S  R  W  P  P  S  T  R  S  L  G  T  L  T  L  W  G
tgggaagctggccctcttccaggccatagggaaggaggttgtggacagggcactggggtg
 W  E  A  G  P  L  P  G  H  R  E  G  G  C  G  Q  G  T  G  V
gagctggctggaggcccaggaggccacggggggcctggtggaccccatccagggcatgcg
 E  L  A  G  G  P  G  G  H  G  G  P  G  G  P  H  P  G  H  A
cgtggcccctgagctggcctgccagcaaagcctcctggaccaggagacgtggtgtgggct
 R  G  P  -  A  G  L  P  A  K  P  P  G  P  G  D  V  V  W  A
gttggagcttgggcccag
 V  G  A  W  A  Q
```

C

Pilot whale (*Globicephala melas*)

>NW_022134900.1:c25207317-25206758 Globicephala melas isolate X-22926-18 unplaced genomic scaffold, ASM654740v1 scaffold47, whole genome shotgun sequence
ATGAACAGCCACGTCTCTCCTCTCAACCTCCTGGTCACCAATGGCACTGAGACGGCTGTCGTCCCTGAGAC
CGTGAAGGCCGTGCTGGGCACCGGCACCCCATCCGGGCCCCAGGCCAGGAGCCTCGCCGGCGTGTATGTGG
AGGCCTCGGGCCAGGCCCAGCGTCTCTACGCTGTCATGAAGCAGGGTCTCCTGCCCTTTGGGCTCGAGCTG
GCTCTGCTGGAGGCCCAGGCGGCCCCGGGCAGGGCCAGCTGCTCCTCGTGTCTGAGGCCCTGCAGCAGGGC
CTGGTGGGCCTGGAGCGGAAGGAGAAGCCGCTGGCCGCCGAGCACGTGGTCACTGGGTACCCTGACCCTCT
GGGGGTGGGAAGCTGGCCCTCTTCCAGGCCATAGGGAAGGAGGTTGTGGACAGGGCACTGGGGTGGAGCTG
GCTGGAGGCCCAGGAGGCCACGGGGGGCCTGGTGGACCCCATCCAGGGCATGCGTGTGGCCCCTGAGCTGG
CCTGCCAGCAAAGCCTCCTGGACCAGGAGACGTGGTGTGGGCTGTTGGAGCTTGGGCCCAGCT

Translation:

```
atgaacagccacgtctctcctctcaacctcctggtcaccaatggcactgagacggctgtc
 M  N  S  H  V  S  P  L  N  L  L  V  T  N  G  T  E  T  A  V
gtccctgagaccgtgaaggccgtgctgggcaccggcaccccatccgggccccaggccagg
 V  P  E  T  V  K  A  V  L  G  T  G  T  P  S  G  P  Q  A  R
agcctcgccggcgtgtatgtggaggcctcgggccaggcccagcgtctctacgctgtcatg
 S  L  A  G  V  Y  V  E  A  S  G  Q  A  Q  R  L  Y  A  V  M
aagcagggtctcctgccctttgggctcgagctggctctgctggaggcccaggcggccccg
 K  Q  G  L  L  P  F  G  L  E  L  A  L  L  E  A  Q  A  A  P
ggcagggccagctgctcctcgtgtctgaggccctgcagcagggcctggtgggcctggagc
```

G R A S C S S C L R P C S R A W W A W S
ggaaggagaagccgctggccgccgagcacgtggtcactgggtaccctgaccctctggggg
G R R S R W P P S T W S L G T L T L W G
tgggaagctggccctcttccaggccatagggaaggaggttgtggacagggcactggggtg
W E A G P L P G H R E G G C G Q G T G V
gagctggctggaggcccaggaggccacgggggcctggtggaccccatccagggcatgcg
E L A G G P G G H G G P G G P H P G H A
tgtggcccctgagctggcctgccagcaaagcctcctggaccaggagacgtggtgtgggct
C G P - A G L P A K P P G P G D V V W A
gttggagcttgggcccag
V G A W A Q

D

Beluga whale (*Delphinapterus leucas*)

>NW_022098049.1:c17309301-17308751 Delphinapterus leucas isolate GAN/ISIS: 26980492/103006 unplaced genomic scaffold, ASM228892v3 scaffoldscaffold58, whole genome shotgun sequence
ATGAACAGCCACGTCTCTCCTCTCAACCTCCTGGTCACCAATGGCACTGAGACGGCTGTCGTCCCTGAGAC
CGTGAAGGCCGTGCTGGGCACCGGCACCCCATCCGGGCCCCAGGCCAAGGCAGCCACGTCCACCCAGGCCA
GGAGCATCGCCGGCGTGTACGCTGTCATGAAGCAGGGTCTCCTGCCCTTTGGGCTCGAGCTGGCTCTGCTG
GAGGCCCAGGCGGCCCCGGGCAGGGCCAGCTGCTCCTCGTGTCTGAGGCCCTGCAGCAGGGCCTGGTGGGC
CTGGAGCGGAAGGAGAAGCCGCTGGCCGCTGAGCGCGTGGTCACTGGGTACCCTGACCCTCTGGGGGTGGG
AAGCTGGCCCTCTTCCAGGCCATAGGGAAGGAGGTTGTGGACAGGGCACTGGGGTGGAGCTGGCTGGAGGC
CCAGGAGGCCACGGGGGGCCTGGTGGACCCCATCCAGGGCATGCGCGTGGCCTCTGAGCTGGCCTGCCAGC
AAAGCCTCCTGGACCGGGAGACATGGTGTGGGCTGTTGGAGCTTGGGCCCAGCT

<u>Translation:</u>
atgaacagccacgtctctcctctcaacctcctggtcaccaatggcactgagacggctgtc
M N S H V S P L N L L V T N G T E T A V
gtccctgagaccgtgaaggccgtgctgggcaccggcaccccatccgggccccaggccaag
V P E T V K A V L G T G T P S G P Q A K
gcagccacgtccacccaggccaggagcatcgccggcgtgtacgctgtcatgaagcagggt
A A T S T Q A R S I A G V Y A V M K Q G
ctcctgccctttgggctcgagctggctctgctggaggcccaggcggccccgggcagggcc
L L P F G L E L A L L E A Q A A P G R A
agctgctcctcgtgtctgaggccctgcagcagggcctggtgggcctggagcggaaggaga
S C S S C L R P C S R A W W A W S G R R
agccgctggccgctgagcgcgtggtcactgggtaccctgaccctctgggggtgggaagct
S R W P L S A W S L G T L T L W G W E A
ggccctcttccaggccatagggaaggaggttgtggacagggcactggggtggagctggct
G P L P G H R E G G C G Q G T G V E L A
ggaggcccaggaggccacgggggcctggtggaccccatccagggcatgcgcgtggcctc
G G P G G H G G P G G P H P G H A R G L
tgagctggcctgccagcaaagcctcctggaccgggagacatggtgtgggctgttggagct
- A G L P A K P P G P G D M V W A V G A
tgggcccag
W A Q

E

Narwhal (*Monodon monoceros*)

```
>NW_021703782.1:114203156-114203706 Monodon monoceros isolate NGI
ecotype Baffin Bay Population unplaced genomic scaffold, NGI_Narwhal_1
Super_Scaffold_5, whole genome shotgun sequence
ATGAACAGCCACGTCTCTCCTCTCAACCTCCTGGTCACCAATGGCACTGAGACGGCTGTCGTCCCTGAGAC
CGTGAAGGCCGTGCTGGGCACCGGCACCCCATCCGGGCCCCAGGCCAAGGCAGCCACGTCCACCCAGGCCA
GGAGCATCGCCGGCGTGTACGCTGTCATGAAGCAGGGTCTCCTGCCCTTTGGGCTCGAGCTGGCTCTGCTG
GAGGCCCAGGCGGCCCCGGGCAGGACCAGCTGCTCCTCGTGTCTGAGGCCGTGCAGCAGGGCCTGGTGGGC
CTGGAGCGGAAGGAGAAGCCGCTGGCCGCTGAGCGCGTGGTCACTGGGTACCCTGACCCTCTGGGGGTGGG
AAGCTGGCCCTCTTCCAGGCCATAGGGAAGGAGATTGTGGACAGGGCACTGGGGTGGAGCTGGCTGGAGGC
CCAGGAGGCCACGGGGGGCCTGGTGGACCCCATCCAGGGCATGCGCGTGGCCTCTGAGCTGGCCTGCCAGC
AAAGCCTCCTGGACCGGGAGACATGGTGTGGGCTGTTGGAGCTTGGGCCCAGCT
```

Translation:

```
atgaacagccacgtctctcctctcaacctcctggtcaccaatggcactgagacggctgtc
 M  N  S  H  V  S  P  L  N  L  L  V  T  N  G  T  E  T  A  V
gtccctgagaccgtgaaggccgtgctgggcaccggcaccccatccgggccccaggccaag
 V  P  E  T  V  K  A  V  L  G  T  G  T  P  S  G  P  Q  A  K
gcagccacgtccacccaggccaggagcatcgccggcgtgtacgctgtcatgaagcagggt
 A  A  T  S  T  Q  A  R  S  I  A  G  V  Y  A  V  M  K  Q  G
ctcctgccctttgggctcgagctggctctgctggaggcccaggcggccccgggcaggacc
 L  L  P  F  G  L  E  L  A  L  L  E  A  Q  A  A  P  G  R  T
agctgctcctcgtgtctgaggccgtgcagcagggcctggtgggcctggagcggaaggaga
 S  C  S  S  C  L  R  P  C  S  R  A  W  W  A  W  S  G  R  R
agccgctggccgctgagcgcgtggtcactgggtaccctgaccctctgggggtgggaagct
 S  R  W  P  L  S  A  W  S  L  G  T  L  T  L  W  G  W  E  A
ggccctcttccaggccatagggaaggagattgtggacagggcactggggtggagctggct
 G  P  L  P  G  H  R  E  G  D  C  G  Q  G  T  G  V  E  L  A
ggaggcccaggaggccacggggggcctggtggaccccatccagggcatgcgcgtggcctc
 G  G  P  G  G  H  G  G  P  G  G  P  H  P  G  H  A  R  G  L
tgagctggcctgccagcaaagcctcctggaccgggagacatggtgtgggctgttggagct
 -  A  G  L  P  A  K  P  P  G  P  G  D  M  V  W  A  V  G  A
tgggcccag
 W  A  Q
```

F

Baiji (*Lipotes vexillifer*)

```
>NW_006791731.1:864401-865100 Lipotes vexillifer unplaced genomic
scaffold, Lipotes_vexillifer_v1 scaffold394, whole genome shotgun
sequence
CAGAGCCTTTCCATCCCCACAGGGAGCAGACGGTGGACCCTGGCTGAGCAAGTTCTGTGCGACGGACAGCC
ACGTCTCTCCTCTCAACCTCCTGGTCACCAGTGGCACTGAGACGGCTGTCGTCCCTGAGACCGTGAAGGCC
AGCACGGGGCACCGGCACCCCATCCGGGCCCCAGGCCAAGGCAGCCACGTCCACCCAGGCCAGGAGCATCG
CCGGGGTGTACGTGGAGGCCTTGGGCCAGGCCCAGCATCTCTACGCTGTCATGAAGCAGGGTCTCCTGCCC
TTTGGGCTCGGGCTGGCTCTGCTAGAGGCCCAGGTGGCCCCGGGCAGGGCCAGCTGCGCCTCGTGTCTGAG
```

GCCCTGCAGCAGGGCCTGGTGGGCCTGGAGCGGAAGGAGAAGCCGCTGGCCGCTGAGTGCGCAGTCACTGG
GTACCCTGACCCTCTGGGGGTGGGAAGCTGGCCCTCTTCCAGGCCATAGGGAAGGAGGTTGTGGACAGGGC
ACTGGGGTGGAGCTGGCTGGAGGCCCAGCGGGCCACGGGGAGCCTGGTGGACCCCATCCAGCGTGTGCGCG
TGGCCCCTGAGCTGGCCTGCCGGCAAAGCCTCCTGGACCAGGACATGGTGTGGGCTGTTGGACCTTGGGCC
TGGCTCAAGTGCCCCAGGCTTCCTGGACCCCAACACGCTGGAGCAGCTGCCATACCGGGAG

Translation:
cagagcctttccatccccacagggagcagacggtggaccctggctgagcaagttctgtgcga
E P F H P H R E Q T V D P G - A S S V R
cggacagccacgtctctcctctcaacctcctggtcaccagtggcactgagacggctgtcg
R T A T S L L S T S W S P V A L R R L S
tccctgagaccgtgaaggccagcacggggcaccggcaccccatccgggccccaggccaag
S L R P - R P A R G T G T P S G P Q A K
gcagccacgtccacccaggccaggagcatcgccggggtgtacgtggaggccttgggccag
A A T S T Q A R S I A G V Y V E A L G Q
gcccagcatctctacgctgtcatgaagcagggtctcctgccctttgggctcgggctggct
A Q H L Y A V M K Q G L L P F G L G L A
ctgctagaggcccaggtggccccgggcagggccagctgcgcctcgtgtctgaggccctgc
L L E A Q V A P G R A S C A S C L R P C
agcagggcctggtgggcctggagcggaaggagaagccgctggccgctgagtgcgcagtca
S R A W W A W S G R R S R W P L S A Q S
ctgggtaccctgaccctctgggggtgggaagctggccctcttccaggccatagggaagga
L G T L T L W G W E A G P L P G H R E G
ggttgtggacagggcactggggtggagctggctggaggcccagcgggccacggggagcct
G C G Q G T G V E L A G G P A G H G E P
ggtggaccccatccagcgtgtgcgcgtggcccctgagctggcctgccggcaaagcctcct
G G P H P A C A R G P - A G L P A K P P
ggaccaggacatggtgtgggctgttggaccttgggcctggctcaagtgccccaggcttcc
G P G H G V G C W T L G L A Q V P Q A S
tggaccccaacacgctggagcagctgccataccggg
W T P T R W S S C H T G

G

Finless porpoise (*Neophocaena asiaeorientalis*)

>NW_020172779.1:c22845918-22845335 Neophocaena asiaeorientalis asiaeorientalis breed wild unplaced genomic scaffold, Neophocaena_asiaeorientalis_V1 scaffold177, whole genome shotgun sequence
ATGAACAGCCACGTCTCTCCTCTCAACCTCCTGGTCACCAATGGCACTGAGACGGCTGTCGTCCCTGAGAC
CGTGAAGGCCGTGCTGGGCACCGGCACCCCATCTGGGCCCCAGGCCAAGGCAGCCACGTCCACCCAGGCCA
GGAGCATCACCGGCGTGTACGCAGAGGCCTCGGGCCAGGCCCAGAGTCTCTACGCTGTCATGAAGCAGGGT
CTCCTGCCCTTTGGGCTCGAGCTGGCTCTGCTGGAGGCCCAGGCGGCCCCGGGCAGGGCCAGCTGCTCCTC
GCGTCTGAGGCCCTGCAGCAGGGCCTGGTGGGCCTGGAGCGGAAGGAGAAGCCGCTGGCCGCCAAGCGCGC
GGTCACTGGGTACCCTGACCCTCTGGGGGTGGGAAGCTGGCCCTCTTCCAGGCCATAGGGAAGGAGGTTGT
GGACAGGGCACTGGGGTGGAGCTGGCTGGAGGCCCAGGAGGCCACGGGGGGCCTGGTGGACCCCATCCAGG
GCATGCGCGTGGCCTCTGAGCTGGCCTGCCAGCAAAGCCTCCTGGACCGGGAGACATGGTGTGGGCTGTTG
GAGCTTGGGCCCAGCT

Translation:

```
atgaacagccacgtctctcctctcaacctcctggtcaccaatggcactgagacggctgtc
 M  N  S  H  V  S  P  L  N  L  L  V  T  N  G  T  E  T  A  V
gtccctgagaccgtgaaggccgtgctgggcaccggcaccccatctgggccccaggccaag
 V  P  E  T  V  K  A  V  L  G  T  G  T  P  S  G  P  Q  A  K
gcagccacgtccacccaggccaggagcatcaccggcgtgtacgcagaggcctcgggccag
 A  A  T  S  T  Q  A  R  S  I  T  G  V  Y  A  E  A  S  G  Q
gcccagagtctctacgctgtcatgaagcagggtctcctgccctttgggctcgagctggct
 A  Q  S  L  Y  A  V  M  K  Q  G  L  L  P  F  G  L  E  L  A
ctgctggaggcccaggcggcccccgggcagggccagctgctcctcgcgtctgaggccctgc
 L  L  E  A  Q  A  A  P  G  R  A  S  C  S  S  R  L  R  P  C
agcagggcctggtgggcctggagcggaaggagaagccgctggccgccaagcgcgcggtca
 S  R  A  W  W  A  W  S  G  R  R  S  R  W  P  P  S  A  R  S
ctgggtaccctgaccctctggggtgggaagctggccctcttccaggccatagggaagga
 L  G  T  L  T  L  W  G  W  E  A  G  P  L  P  G  H  R  E  G
ggttgtggacagggcactggggtggagctggctggaggcccaggaggccacggggggcct
 G  C  G  Q  G  T  G  V  E  L  A  G  G  P  G  G  H  G  G  P
ggtggaccccatccagggcatgcgcgtggcctctgagctggcctgccagcaaagcctcct
 G  G  P  H  P  G  H  A  R  G  L  -  A  G  L  P  A  K  P  P
ggaccgggagacatggtgtgggctgttggagcttgggcccag
 G  P  G  D  M  V  W  A  V  G  A  W  A  Q
```

H

Vaquita (*Phocoena sinus*)

```
>NC_045779.1:c78699436-78697937 Phocoena sinus isolate mPhoSin1
chromosome 17, mPhoSin1.pri, whole genome shotgun sequence
ATGAACAGCCACGTCTCTCCTCTCAACCTCCTGGTCACCAATGGCACTGAGACGGCTGTCGTCCCTGAGAC
CGTGAAGGCCGTGCTGGGCACCGGCACCCCATCTGGACCCCAGGCCAAGGCAGCCACGTCCACCCAGGCCA
GGAGCATCGCCGGCGTGTACGCAGAGGCCTCGGGCCAGGCCCAGAGTCTCTACGCTGTCATGAAGCAGGGT
CTCCTGCCCTTTGGGCTCGAGCTGGCTCTGCTGGAGGCCCAGGCGGCCCCGGGCAGGGCCAGCTGCTCCTC
GCGTCTGAGGCCCTGCAGCAGGGCCTGGTGGGCCTGGAGCGGAAGGAGAAGCCGCTGGCCGCCGAGCGCGC
GGTCACTGGGTACCCTGACCCTCTGGGGGTGGGAAGCTGGCCCTCTTCCAGGCCATAGGGAAGGAGGTTGT
GGACAGGGCACTGGGGTGGAGCTGGCTGGAGGCCCAGGAGGGCCACGGGGGGGCCTGGTGGACCCCATCCA
GGGCATGCGCGTGGCCTCTGAGCTGGCCTGCCAGCAAAGCCTCCTGGACCGGGAGACATGGTGTGGGCTGT
TGGAGCTTGGGCCCAGCTCAAGTGCCCCAGGCTTCCTGGACCCCGACACGCCGGAGCAGCTTGCCATACCG
GGAGCTGCTAGGCATGTGTGTGTGCAGGCCCCCAGCACGGGGCCTGGGCCTACTGCCCCTCAAGATCACTT
TCCGTACGCTGAGTGGGGCACCGAGCTTTGGCCAGGCCGCTGGAGGTGGGGGTCCTGGACGCGGGAGACGG
TCCAGGCCTGCGGGAGGGCAGGCTGGCAGTGCCGGATGTGGGTGCACGCACCGAGGTGCAGCGCCACCCGC
AGGGCACCGGCGGTGTGGCAGGGCTCGTCCTGCTGCTGCAGGGCACAAGAGCTTCTTCCAGGCCTAGCCGA
GCACCTGCTCCCACGGGCGCCACGCTTCCGTCCAGGAGGCTCAGGACGCCACCCGCATGCTGGTGGACCCA
GCCACCGCGCCGGCAGCTGTGGCTGGAATGAGGCGGTCAGGGCAGGCCTGTTTGGACCAGAGCTCCACAGG
CAGCTCCTGGCAGCAGAGCAGGTGGTGACGGGGTATCATGGCCCCTTCGGTAGGTGGCATCCGAATCCCCT
ATTCCAGGCCACGAAGAGGGGAGCTGGTGGACAGGCCTCCGGTGCTGAGGCTCTTGGTTGCCCAGCTGGCT
ACAGGCAGGCTCCGGCCTGCCCTTGGAGGCCACCCTGCACTTCGGCTGCCTGGACAGACTCAGCAGCATCT
CTCGCAGGCGGCTGGCTTTCTCGTAGCCCCGGCAGCAGGAGAGCCTCAGCTGTGGGCAGCTGCTGGCCCAC
TGTGTCACCGACCCAGAGACGGGGCTGGGCCTTCCTGCCATCTCAGTGGGGAGCCCTGGAGCGGAGCCACA
GGGGGGCCCCCACTCACTGAGGCACAGCACCTGGCAGACCTTGAGAGCTGCCGTGGCCACCGTCTCCAATG
GGCAAGTTC
```

Translation:

```
atgaacagccacgtctctcctctcaacctcctggtcaccaatggcactgagacggctgtc
 M  N  S  H  V  S  P  L  N  L  L  V  T  N  G  T  E  T  A  V
gtccctgagaccgtgaaggccgtgctgggcaccggcaccccatctggaccccaggccaag
 V  P  E  T  V  K  A  V  L  G  T  G  T  P  S  G  P  Q  A  K
gcagccacgtccacccaggccaggagcatcgccggcgtgtacgcagaggcctcgggccag
 A  A  T  S  T  Q  A  R  S  I  A  G  V  Y  A  E  A  S  G  Q
gcccagagtctctacgctgtcatgaagcagggtctcctgccctttgggctcgagctggct
 A  Q  S  L  Y  A  V  M  K  Q  G  L  L  P  F  G  L  E  L  A
ctgctggaggcccaggcggcccccgggcagggccagctgctcctcgcgtctgaggccctgc
 L  L  E  A  Q  A  A  P  G  R  A  S  C  S  S  R  L  R  P  C
agcagggcctggtgggcctggagcggaaggagaagccgctggccgccgagcgcgcggtca
 S  R  A  W  W  A  W  S  G  R  R  S  R  W  P  P  S  A  R  S
ctgggtaccctgaccctctgggggtgggaagctggccctcttccaggccatagggaagga
 L  G  T  L  T  L  W  G  W  E  A  G  P  L  P  G  H  R  E  G
ggttgtggacagggcactggggtggagctggctggaggcccaggagggccacgggggggc
 G  C  G  Q  G  T  G  V  E  L  A  G  G  P  G  G  P  R  G  G
ctggtggaccccatccagggcatgcgcgtggcctctgagctggcctgccagcaaagcctc
 L  V  D  P  I  Q  G  M  R  V  A  S  E  L  A  C  Q  Q  S  L
ctggaccgggagacatggtgtgggctgttggagcttgggcccagctcaagtgccccaggc
 L  D  R  E  T  W  C  G  L  L  E  L  G  P  S  S  S  A  P  G
ttcctggaccccgacacgccggagcagcttgccataccgggagctgctaggcatgtgtgt
 F  L  D  P  D  T  P  E  Q  L  A  I  P  G  A  A  R  H  V  C
gtgcaggcccccagcacggggcctgggcctactgcccctcaagatcactttccgtacgct
 V  Q  A  P  S  T  G  P  G  P  T  A  P  Q  D  H  F  P  Y  A
gagtggggcaccgagctttggccaggccgctggaggtgggggtcctggacgcgggagacg
 E  W  G  T  E  L  W  P  G  R  W  R  W  G  S  W  T  R  E  T
gtccaggcctgcgggagggcaggctggcagtgccggatgtgggtgcacgcaccgaggtgc
 V  Q  A  C  G  R  A  G  W  Q  C  R  M  W  V  H  A  P  R  C
agcgccacccgcagggcaccggcggtgtggcagggctcgtcctgctgctgcagggcacaa
 S  A  T  R  R  A  P  A  V  W  Q  G  S  S  C  C  C  R  A  Q
gagcttcttccaggcctagccgagcacctgctcccacgggcgccacgcttccgtccagga
 E  L  L  P  G  L  A  E  H  L  L  P  R  A  P  R  F  R  P  G
ggctcaggacgccacccgcatgctggtggacccagccaccgcgccggcagctgtggctgg
 G  S  G  R  H  P  H  A  G  G  P  S  H  R  A  G  S  C  G  W
aatgaggcggtcagggcaggcctgtttggaccagagctccacaggcagctcctggcagca
 N  E  A  V  R  A  G  L  F  G  P  E  L  H  R  Q  L  L  A  A
gagcaggtggtgacggggtatcatggccccttcggtaggtggcatccgaatcccctattc
 E  Q  V  V  T  G  Y  H  G  P  F  G  R  W  H  P  N  P  L  F
caggccacgaagaggggagctggtggacaggcctccggtgctgaggctcttggttgccca
 Q  A  T  K  R  G  A  G  G  Q  A  S  G  A  E  A  L  G  C  P
gctggctacaggcaggctccggcctgcccttggaggccaccctgcacttcggctgcctgg
 A  G  Y  R  Q  A  P  A  C  P  W  R  P  P  C  T  S  A  A  W
acagactcagcagcatctctcgcaggcggctggctttctcgtagccccggcagcaggaga
 T  D  S  A  A  S  L  A  G  G  W  L  S  R  S  P  G  S  R  R
gcctcagctgtgggcagctgctggcccactgtgtcaccgacccagagacggggctgggcc
 A  S  A  V  G  S  C  W  P  T  V  S  P  T  Q  R  R  G  W  A
ttcctgccatctcagtggggagccctggagcggagccacaggggggcccccactcactga
 F  L  P  S  Q  W  G  A  L  E  R  S  H  R  G  A  P  T  H  -
ggcacagcacctggcagaccttgagagctgccgtggccaccgtctccaatgggcaagttc
 G  T  A  P  G  R  P  -  E  L  P  W  P  P  S  P  M  G  K  F
```

Predicted amino acid sequence (FASTA format):
> Phocoena_sinus_EPPK1
MNSHVSPLNLLVTNGTETAVVPETVKAVLGTGTPSGPQAKAATSTQARSIAGVYAEASGQAQSLYAVMKQG
LLPFGLELALLEAQAAPGRASCSSRLRPCSRAWWAWSGRRSRWPPSARSLGTLTLWGWEAGPLPGHREGGC
GQGTGVELAGGPGGPRGGLVDPIQGMRVASELACQQSLLDRETWCGLLELGPSSSAPGFLDPDTPEQLAIP
GAARHVCVQAPSTGPGPTAPQDHFPYAEWGTELWPGRWRWGSWTRETVQACGRAGWQCRMWVHAPRCSATR
RAPAVWQGSSCCCRAQELLPGLAEHLLPRAPRFRPGGSGRHPHAGGPSHRAGSCGWNEAVRAGLFGPELHR
QLLAAEQVVTGYHGPFGRWHPNPLFQATKRGAGGQASGAEALGCPAGYRQAPACPWRPPCTSAAWTDSAAS
LAGGWLSRSPGSRRASAVGSCWPTVSPTQRRGWAFLPSQWGALERSHRGAPTH

Amino acid sequence alignment:

```
                            1                                                   Plakin repeat domain 1
Human epiplakin N-terminus  MSGHTLPPLPVPGTNSTEQASVPRAMAATLGAGTPPRPQA--------RSIAGVYVEASGQAQSVYAAMEQGLLPAGLGQ
Vaquita epiplakin           MNSH-VSPLNLLVTNGTETAVVPETVKAVLGTGTPSGPQAKAATSTQARSIAGVYAEASGQAQSLYAVMKQGLLPFGLEL

                            81
Human epiplakin N-terminus  ALLEAQAATGGLVDLARGQLLPVSKALQQGLVGLELKEKLLAAERATTGYPDPYGGEKLALFQAIGKEVVDRALGQSWLE
Vaquita epiplakin           ALLEAQAAPGRASCSSR--LRPCSRAWW----AWSGRRSRWPPSARSLGTLTLWGWEAGPLPGHREGGCGQGTGVELAGG

                            161
Human epiplakin N-terminus  VQLATGGLVDPAQGVLVAPEPACHQGLLDRETWHKLSELEPGTGDLRFLDPNTLERLTYHQLLER-CVRAPGSGLALLPL
Vaquita epiplakin           PGGPRGGLVDPIQGMRVASELACQQSLLDRETWCGLLELGPSSSAPGFLDPDTPEQLAIPGAARHVCVQAPSTGPGPTAP

                            241                                                         Plakin repeat domain 2
Human epiplakin N-terminus  KITFR-SMGGAVSAAELLEVGILDEQAVQGL-REGRLAAVDVSA-RAEVRRYLEGTGSVAGVVLLPEGHKKSFFQAATEH
Vaquita epiplakin           QDHFPYAEWGTELWPGRWRWGSWTRETVQACGRAGWQCRMWVHAPRCSATRRAPAVWQGSSCCC----RAQELLPGLAEH

                            321
Human epiplakin N-terminus  LLPMGTALPLLEAQAATHTLVDPITGQRLWVDEAVRAGLVSPELHEQLLVAEQAVTGHHDPFSG-SQIPLFQAMKKGLVD
Vaquita epiplakin           LLPRAPRFRPGGSGRHPHAGGPSHRAGSCGWNEAVRAGLFGPELHRQLLAAEQVVTGYHGPFGRWHPNPLFQATKRGAGG

                            401
Human epiplakin N-terminus  RPLALRLLDAQLATGGLVCPARRLRLPLEAALRCGCLDEDTQRQLSQAGSFSDGTHGGLRYEQLLALCVTDPETGLAFLP
Vaquita epiplakin           QASGAEALGCP--AGYRQAPACPWRPPCTSAAWTDSAASLAGGWLSR----SPGSRRASAVGSCWPTVSPTQRRGWAFLP

                            481
Human epiplakin N-terminus  LSGGPRGGEPQGPPFIKYSTRQALSTATATVSVGKFRGRPVSLWELLFSEAISSEQRAMLAQQYQEGTLSVEKLAAKLSA
Vaquita epiplakin           SQWGALERSHRGAPTH*---------------------------------------------------------------
```

I

Minke whale (*Balaenoptera acutorostrata*)

>NW_006725399.1:1961851-1962449 Balaenoptera acutorostrata scammoni unplaced genomic scaffold, BalAcu1.0 scaffold114, whole genome shotgun sequence
ATGAGCAGCCACACCTCTCCTCTCCACCTCCTGGTCACCAGTGGCACTGAGACGGCTGTCGTCCCCGAGAC
CGTGACGGCCGTGCTGGGCACCGGCACCCCGTCCGGGCCCCAGGCCAAGGCAGCCACGTCCACGCAGGCCA
GGAGCATCACGGGGTGTACGTGGAGGCCTCGGGCCAGGCCCAGCGTCTCTGCGCTGTCATGAGGCAGGGTC
TCCTGCCCTTTGGGCTCGGGCTGGCTCTGCTGGAGGCCCAGGCGGCCACCGGGGGCCGCACGGACCCCGGG
CAGGGCCAGCTGCTCCTCGTGTCTGAGGCCCTGCAGCAGGCCTGGTGGGCCTGGAGCGGAAGGAGAAGCTG
CTGGCCGCTGAGCGCGCCGTCACCGGGTACCCTGACCCTCTGGGGGTGGGAAGCTGGCCCTCTTCCAGGCC
ATAGGGAAGGAGGTTGTGGACAGGGCACTGGGCTGGAGCTGCGGAGGCCCAGCTGGCCACGGGGGGCCTGG
TGGACCCCATCCAGGGCGTGCGCGTGGCCCCTGAGCTGGCCTGCCAGCAAAGCCTCTTGGCCCAGGAGACA
TGGTGTGGGTTGCTGGAGCTTGGGCCCGGCT

Translation:

```
atgagcagccacacctctcctctccacctcctggtcaccagtggcactgagacggctgtc
 M  S  S  H  T  S  P  L  H  L  L  V  T  S  G  T  E  T  A  V
```

```
gtccccgagaccgtgacggccgtgctgggcaccggcaccccgtccgggccccaggccaag
 V  P  E  T  V  T  A  V  L  G  T  G  T  P  S  G  P  Q  A  K
gcagccacgtccacgcaggccaggagcatcacggggtgtacgtggaggcctcgggccagg
 A  A  T  S  T  Q  A  R  S  I  T  G  C  T  W  R  P  R  A  R
cccagcgtctctgcgctgtcatgaggcagggtctcctgccctttgggctcgggctggctc
 P  S  V  S  A  L  S  -  G  R  V  S  C  P  L  G  S  G  W  L
tgctggaggcccaggcggccaccgggggccgcacggaccccgggcagggccagctgctcc
 C  W  R  P  R  R  P  P  G  A  A  R  T  P  G  R  A  S  C  S
tcgtgtctgaggccctgcagcaggcctggtgggcctggagcggaaggagaagctgctggc
 S  C  L  R  P  C  S  R  P  G  G  P  G  A  E  G  E  A  A  G
cgctgagcgcgccgtcaccgggtaccctgaccctctgggggtgggaagctggccctcttc
 R  -  A  R  R  H  R  V  P  -  P  S  G  G  G  K  L  A  L  F
caggccatagggaaggaggttgtggacagggcactgggctggagctgcggaggcccagct
 Q  A  I  G  K  E  V  V  D  R  A  L  G  W  S  C  G  G  P  A
ggccacgggggcctggtggaccccatccagggcgtgcgcgtggcccctgagctggcctg
 G  H  G  G  P  G  G  P  H  P  G  R  A  R  G  P  -  A  G  L
ccagcaaagcctcttggcccaggagacatggtgtgggttgctggagcttgggcccgg
 P  A  K  P  L  G  P  G  D  M  V  W  V  A  G  A  W  A  R
```

J

Sperm whale (*Physeter catodon*)

Note: The following sequence is homologous to an internal segment of the human *EPPK1* gene. A sequence homologous to the start of the coding sequence of human *EPPK1* is not present in the genome of the sperm whale (status 25 November 2021).

```
>NW_021146471.1:c23000-22001 Physeter catodon isolate SW-GA unplaced
genomic scaffold, ASM283717v2 random_1047, whole genome shotgun
sequence
GAGGGGACACAGCCGGGCTGAGGTGGGAGACTTGGGACAGAGGGCAGGGGTCTGGCTGAAGCCGGTGGG
GAGCCAGGACGGAGCAGAGGCCAGGGCAGCAGCGGGTGAAGGTGGGGGAGGATTTGGAGGGGTCACTGGG
GAGGGAGAAGAGCACGAATGCATGGAGACTGCCCAAGGGCAGGGCCGGGACGGGGCCTTTGGGAGCCAGA
AGGAGGGCCGGTCGTTGGCCGCGAACCGCCAAAAAAAAGGTTTGTGGATCCCAACACGCAGGAGAAGGTG
ACTTACCAGGAGCTGCAGGAGAGGAGCCACCCGGAAGAGGGCACGGACTGGGCCCTGTTCCCCAGGGTCA
GCGGCAGACGGGACTCCAGCTTCATCAACAAGGCAACCAGAAGGGCCCCCACCCCCAAAGCCGAGCGGGT
GGATGTCACAGTGGGAAGGTTCAAGGGCCAGAGACCATTGGTCTGGGAACTGCTGAACTCAGAATACTTG
CCAGAGGACAAAAAACGTGAGTTAGTAGTAAAATACAAGAGGGACACGACACATGCACTAGAGAAGGTAG
TAAAAGTTATTTTCGAGATAATTGATGAGAAGGAAAAGAGCAATAGACAGTTAATGGTTCAGAGGGGTCA
GGAGGCAGATCACAGCCTCTGAACTCCTGCAACCAGGCATCATCACTGAGGAGGCCCGGAGGGAGGGCGG
GCCACCGTGGAGGACGCCGAAAGAGCCAGGGGGTAAAGCGCTACCTGGAGGGCACCAGCTGCATCGCGGG
CACGCTGGTGCCCGCCAAGGACGAGCCCGGGCGCCAGGAGAAGAGGAGGATCTACCAGGCCATGTGGAAG
GGCATCCTGAGGCCGGGCACAGCCCTGGTGCTGCTGGAGGCACAGGCGGCCACCGGCTTCGTCACCTACC
CCCACGGAGAACCGGAAGCTGACTGTGCAGGGGGCGTTTGCTGCCGGGATGTTCAGCAGTGAAACCTACC
AGAAGCTGCTGTCGGCCGAG
```

Translation:

5'3' Frame 1

```
gaggggacacagccgggctgaggtgggagacttgggacagagggcaggggtctggctga
 E  G  D  T  A  G  L  R  W  E  T  W  D  R  G  Q  G  S  G  -
```

```
agccggtggggagccaggacggagcagaggccagggcagcagcgggtgaaggtgggggag
 S  R  W  G  A  R  T  E  Q  R  P  G  Q  Q  R  V  K  V  G  E
gatttggaggggtcactggggagggagaagagcacgaatgcatggagactgcccaagggc
 D  L  E  G  S  L  G  R  E  K  S  T  N  A  W  R  L  P  K  G
agggccgggacggggcctttgggagccagaaggagggccggtcgttggccgcgaaccgcc
 R  A  G  T  G  P  L  G  A  R  R  R  A  G  R  W  P  R  T  A
aaaaaaaaggtttgtggatcccaacacgcaggagaaggtgacttaccaggagctgcagga
 K  K  K  V  C  G  S  Q  H  A  G  E  G  D  L  P  G  A  A  G
gaggagccacccggaagagggcacggactgggccctgttccccagggtcagcggcagacg
 E  E  P  P  G  R  G  H  G  L  G  P  V  P  Q  G  Q  R  Q  T
ggactccagcttcatcaacaaggcaaccagaagggcccccacccccaaagccgagcgggt
 G  L  Q  L  H  Q  Q  G  N  Q  K  G  P  H  P  Q  S  R  A  G
ggatgtcacagtgggaaggttcaagggccagagaccattggtctgggaactgctgaactc
 G  C  H  S  G  K  V  Q  G  P  E  T  I  G  L  G  T  A  E  L
agaatacttgccagaggacaaaaaacgtgagttagtagtaaaatacaagagggacacgac
 R  I  L  A  R  G  Q  K  T  -  V  S  S  K  I  Q  E  G  H  D
acatgcactagagaaggtagtaaaagttattttcgagataattgatgagaaggaaaagag
 T  C  T  R  E  G  S  K  S  Y  F  R  D  N  -  -  E  G  K  E
caatagacagttaatggttcagaggggtcaggaggcagatcacagcctctgaactcctgc
 Q  -  T  V  N  G  S  E  G  S  G  G  R  S  Q  P  L  N  S  C
aaccaggcatcatcactgaggaggcccggagggagggcgggccaccgtggaggacgccga
 N  Q  A  S  S  L  R  R  P  G  G  R  A  G  H  R  G  G  R  R
aagagccaggggtaaagcgctacctggagggcaccagctgcatcgcgggcacgctggtg
 K  S  Q  G  V  K  R  Y  L  E  G  T  S  C  I  A  G  T  L  V
cccgccaaggacgagcccgggcgccaggagaagaggaggatctaccaggccatgtggaag
 P  A  K  D  E  P  G  R  Q  E  K  R  R  I  Y  Q  A  M  W  K
ggcatcctgaggccgggcacagccctggtgctgctggaggcacaggcggccaccggcttc
 G  I  L  R  P  G  T  A  L  V  L  L  E  A  Q  A  A  T  G  F
gtcacctacccccacggagaaccggaagctgactgtgcagggggcgtttgctgccgggat
 V  T  Y  P  H  G  E  P  E  A  D  C  A  G  G  V  C  C  R  D
gttcagcagtgaaacctaccagaagctgctgtcggccga
 V  Q  Q  -  N  L  P  E  A  A  V  G  R
```

5'3' Frame 2

```
gagggggacacagccgggctgaggtgggagacttgggacagagggcaggggtctggctgaa
  R  G  T  Q  P  G  -  G  G  R  L  G  T  E  G  R  G  L  A  E
gccggtggggagccaggacggagcagaggccagggcagcagcgggtgaaggtgggggagg
 A  G  G  E  P  G  R  S  R  G  Q  G  S  S  G  -  R  W  G  R
atttggaggggtcactggggagggagaagagcacgaatgcatggagactgcccaagggca
 I  W  R  G  H  W  G  G  R  R  A  R  M  H  G  D  C  P  R  A
gggccgggacggggcctttgggagccagaaggagggccggtcgttggccgcgaaccgcca
 G  P  G  R  G  L  W  E  P  E  G  G  P  V  V  G  R  E  P  P
aaaaaaaggtttgtggatcccaacacgcaggagaaggtgacttaccaggagctgcaggag
 K  K  R  F  V  D  P  N  T  Q  E  K  V  T  Y  Q  E  L  Q  E
aggagccacccggaagagggcacggactgggccctgttccccagggtcagcggcagacgg
 R  S  H  P  E  E  G  T  D  W  A  L  F  P  R  V  S  G  R  R
gactccagcttcatcaacaaggcaaccagaagggcccccacccccaaagccgagcgggtg
 D  S  S  F  I  N  K  A  T  R  R  A  P  T  P  K  A  E  R  V
gatgtcacagtgggaaggttcaagggccagagaccattggtctgggaactgctgaactca
 D  V  T  V  G  R  F  K  G  Q  R  P  L  V  W  E  L  L  N  S
gaatacttgccagaggacaaaaaacgtgagttagtagtaaaatacaagagggacacgaca
 E  Y  L  P  E  D  K  K  R  E  L  V  V  K  Y  K  R  D  T  T
catgcactagagaaggtagtaaaagttattttcgagataattgatgagaaggaaaagagc
```

```
 H  A  L  E  K  V  V  K  V  I  F  E  I  I  D  E  K  E  K  S
aatagacagttaatggttcagaggggtcaggaggcagatcacagcctctgaactcctgca
 N  R  Q  L  M  V  Q  R  G  Q  E  A  D  H  S  L  -  T  P  A
accaggcatcatcactgaggaggcccggagggagggcgggccaccgtggaggacgccgaa
 T  R  H  H  H  -  G  G  P  E  G  G  R  A  T  V  E  D  A  E
agagccagggggtaaagcgctacctggagggcaccagctgcatcgcggcacgctggtgc
 R  A  R  G  -  S  A  T  W  R  A  P  A  A  S  R  A  R  W  C
ccgccaaggacgagcccgggcgccaggagaagaggaggatctaccaggccatgtggaagg
 P  P  R  T  S  P  G  A  R  R  R  G  G  S  T  R  P  C  G  R
gcatcctgaggccgggcacagccctggtgctgctggaggcacaggcggccaccggcttcg
 A  S  -  G  R  A  Q  P  W  C  C  W  R  H  R  R  P  P  A  S
tcacctacccccacggagaaccggaagctgactgtgcaggggcgtttgctgccgggatg
 S  P  T  P  T  E  N  R  K  L  T  V  Q  G  A  F  A  A  G  M
ttcagcagtgaaacctaccagaagctgctgtcggccgag
 F  S  S  E  T  Y  Q  K  L  L  S  A  E
```

5'3' Frame 3

```
gaggggacacagccgggctgaggtgggagacttgggacagagggcaggggtctggctgaag
   G  G  H  S  R  A  E  V  G  D  L  G  Q  R  A  G  V  W  L  K
ccggtggggagccaggacggagcagaggccagggcagcagcgggtgaaggtggggagga
 P  V  G  S  Q  D  G  A  E  A  R  A  A  A  G  E  G  G  G  G
tttggaggggtcactggggagggagaagagcacgaatgcatggagactgcccaagggcag
 F  G  G  V  T  G  E  G  E  E  H  E  C  M  E  T  A  Q  G  Q
ggccgggacggggcctttgggagccagaaggagggccggtcgttggccgcgaaccgccaa
 G  R  D  G  A  F  G  S  Q  K  E  G  R  S  L  A  A  N  R  Q
aaaaaaggtttgtggatcccaacacgcaggagaaggtgacttaccaggagctgcaggaga
 K  K  G  L  W  I  P  T  R  R  R  R  -  L  T  R  S  C  R  R
ggagccacccggaagagggcacggactgggccctgttccccagggtcagcggcagacggg
 G  A  T  R  K  R  A  R  T  G  P  C  S  P  G  S  A  A  D  G
actccagcttcatcaacaaggcaaccagaagggcccccacccccaaagccgagcgggtgg
 T  P  A  S  S  T  R  Q  P  E  G  P  P  P  P  K  P  S  G  W
atgtcacagtgggaaggttcaagggccagagaccattggtctgggaactgctgaactcag
 M  S  Q  W  E  G  S  R  A  R  D  H  W  S  G  N  C  -  T  Q
aatacttgccagaggacaaaaaacgtgagttagtagtaaaatacaagagggacacgacac
 N  T  C  Q  R  T  K  N  V  S  -  -  -  N  T  R  G  T  R  H
atgcactagagaaggtagtaaaagttatttcgagataattgatgagaaggaaaagagca
 M  H  -  R  R  -  -  K  L  F  S  R  -  L  M  R  R  K  R  A
atagacagttaatggttcagaggggtcaggaggcagatcacagcctctgaactcctgcaa
 I  D  S  -  W  F  R  G  V  R  R  Q  I  T  A  S  E  L  L  Q
ccaggcatcatcactgaggaggcccggagggagggcgggccaccgtggaggacgccgaaa
 P  G  I  I  T  E  E  A  R  R  E  G  G  P  P  W  R  T  P  K
gagccagggggtaaagcgctacctggagggcaccagctgcatcgcggcacgctggtgcc
 E  P  G  G  K  A  L  P  G  G  H  Q  L  H  R  G  H  A  G  A
cgccaaggacgagcccgggcgccaggagaagaggaggatctaccaggccatgtggaaggg
 R  Q  G  R  A  R  A  P  G  E  E  E  D  L  P  G  H  V  E  G
catcctgaggccgggcacagccctggtgctgctggaggcacaggcggccaccggcttcgt
 H  P  E  A  G  H  S  P  G  A  A  G  G  T  G  G  H  R  L  R
cacctacccccacggagaaccggaagctgactgtgcaggggcgtttgctgccgggatgt
 H  L  P  P  R  R  T  G  S  -  L  C  R  G  R  L  L  P  G  C
tcagcagtgaaacctaccagaagctgctgtcggccg
 S  A  V  K  P  T  R  S  C  C  R  P
```

```
Predicted amino acid sequence (FASTA format):
> Physeter_catodon_EPPK1
MHGDCPRAGPGRGLWEPEGGPVVGREPPKKRFVDPNTQEKVTYQELQERSHPEEGTDWALFPRVSGRRDSS
FINKATRRAPTPKAERVDVTVGRFKGQRPLVWELLNSEYLPEDKKRELVVKYKRDTTHALEKVVKVIFEII
DEKEKSNRQLMVQRGQEADHSL
```

BLASTp Sperm whale EPPK1 (theoretical) versus human EPPK1:

```
epiplakin [Homo sapiens]: NP_112598.3 (Length: 5088)

Sperm whale EPPK1  29    KKRFVDPNTQEKVTYQELQERSHPEEGTDWALFPRVSGRRDSSFINKATRRAPTPKAERV  88
                         +KRFVDPNTQEKV+Y+ELQER  P+E T W LFP     RDS  I+  TRRA   +AE+V
Human EPPK1        2051  RKRFVDPNTQEKVSYRELQERCRPQEDTGWLLFPVNKAARDSEHIDDETRRAL--EAEQV  2108

Sperm whale EPPK1  89    DVTVGRFKGQRPLVWELLNSEYLPEDKKRELVVKYKRDTTHALEKVVKVIFEIIDEKEKS  148
                         ++TVGRF+GQ+P +W LLNSEY+ E+KK +LV  Y+  T  AL+ V ++I E+I+++E S
Human EPPK1        2109  EITVGRFRGQKPTLWALLNSEYVTEEKKLQLVRMYRTHTRRALQTVAQLILELIEKQETS  2168

Sperm whale EPPK1  149   NRQLMVQ  155
                         N+ L  Q
Human EPPK1        2169  NKHLWFQ  2175
```

Supplementary Figure S1. Analysis of *EPPK1* nucleotide sequences in cetaceans. Nucleotide sequences of *EPPK1* genes were downloaded from GenBank under the accession numbers as indicated in the subject line of FASTA sequences. Nucleotide numbers corresponding to the start and end of the sequence range are indicated, with "c" indicating that the sequence of the complementary strand is shown. The sequences in panels **A**-**E** and **F**-**I** include the region from the conserved start codon to the first in-frame stop codon. In the EPPK1 gene of the baiji (*Lipotes vexillifer*) (**F**) the ancestral start codon is destroyed by a mutation (highlighted by red shading). Therefore, a downstream ATG was used as the start codon of a theoretical coding sequence. The genome of the sperm whale (*Physeter catodon*) (**J**) does not contain a region homologous to the ancestral start of the *EPPK1* coding sequence. Assuming other starts of coding sequence, only short translation products are possible due to in-frame stop codons. An example is shown in panel **J**. Note that *EPPK1* of the vaquita (**H**) contains an open reading for a relatively long protein (479 amino acid residues) which, however, displays less than 40% sequence identity with the N-terminus of human epiplakin (total length: 5088 amino acid residues). Predicted coding sequences are highlighted by yellow shading and the corresponding amino acid sequences are shown below the nucleotide sequences. Stop codons are shown by white fonts on black background.

Human (*Homo sapiens*) EPPK1 (NP_112598.3)

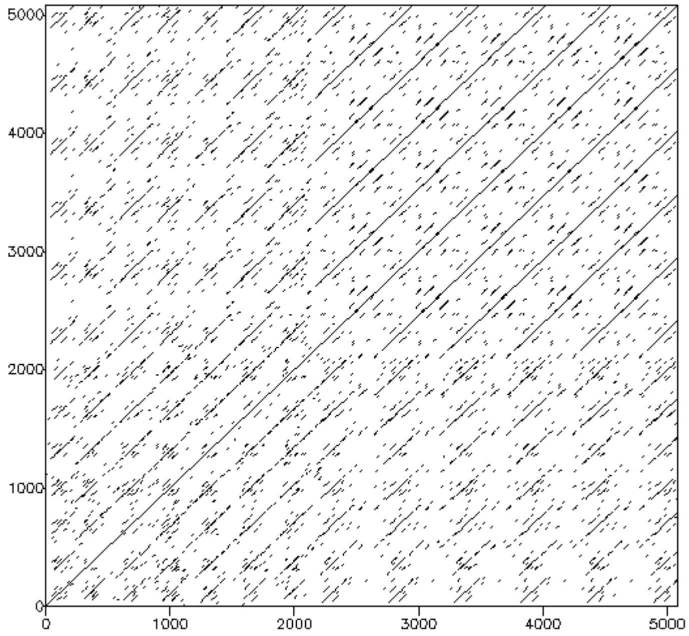

Cattle (*Bos taurus*) EPPK1 (XP_003586920.3)

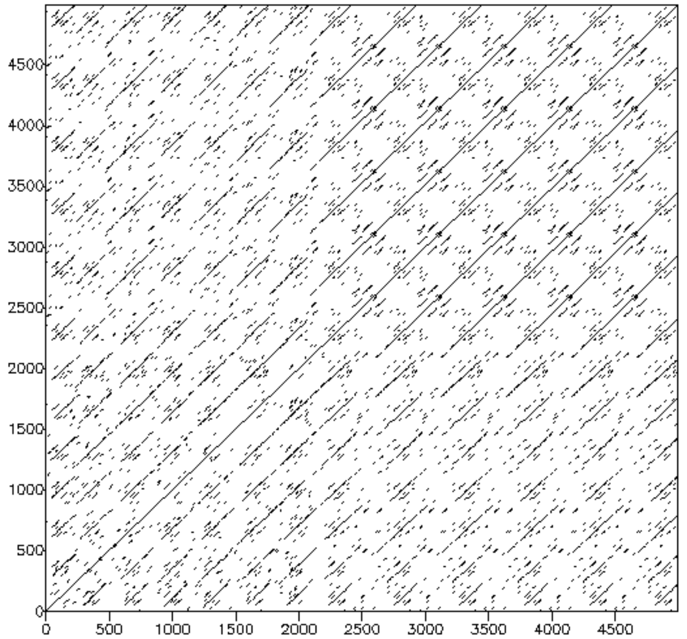

Opossum (*Monodelphis domestica*) EPPK1 (XP_007488766.1)

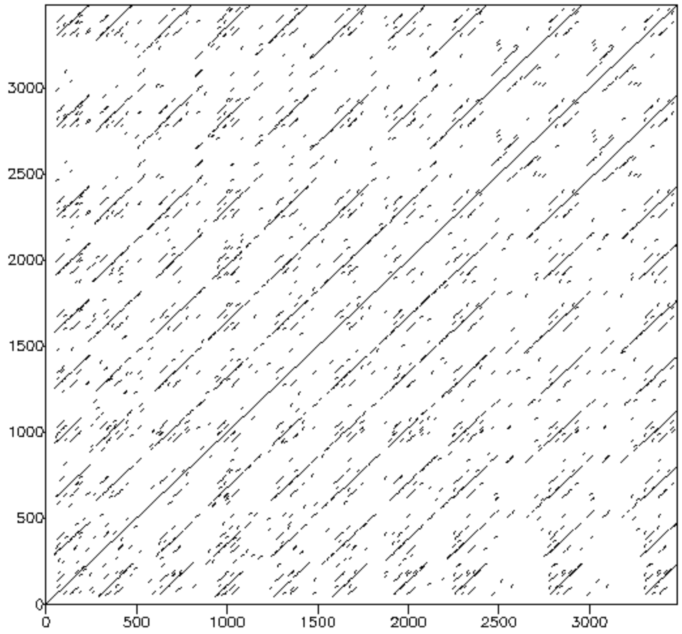

Platypus (*Ornithorhynchus anatinus*) EPPK1 (XP_028919575.1)

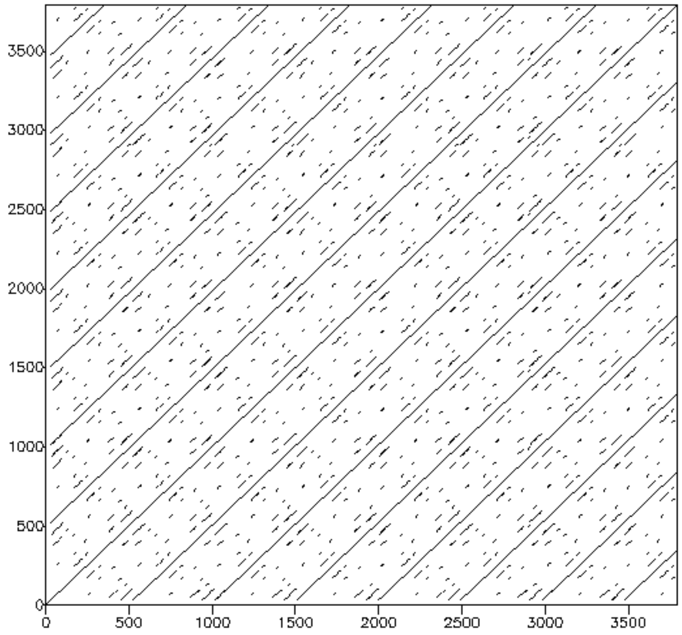

Zebra finch (*Taeniopygia guttata*) EPPK1 (XP_041569977.1)

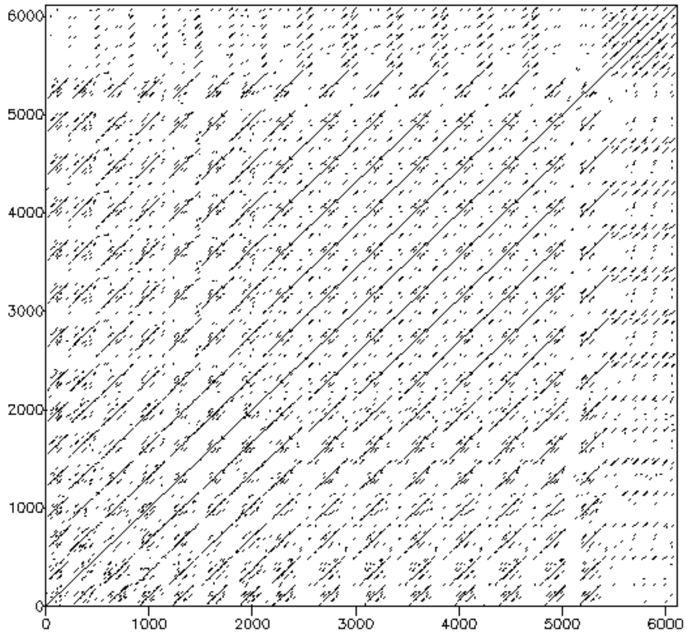

Kiwi (*Apteryx rowi*) EPPK1 (XP_025944119.1)

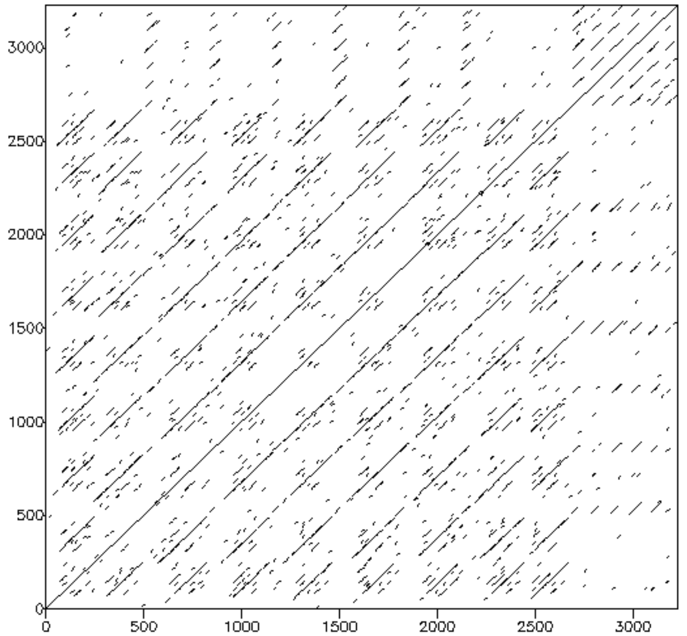

Lizard (*Podarcis muralis*) EPPK1 (XP_028591693.1)

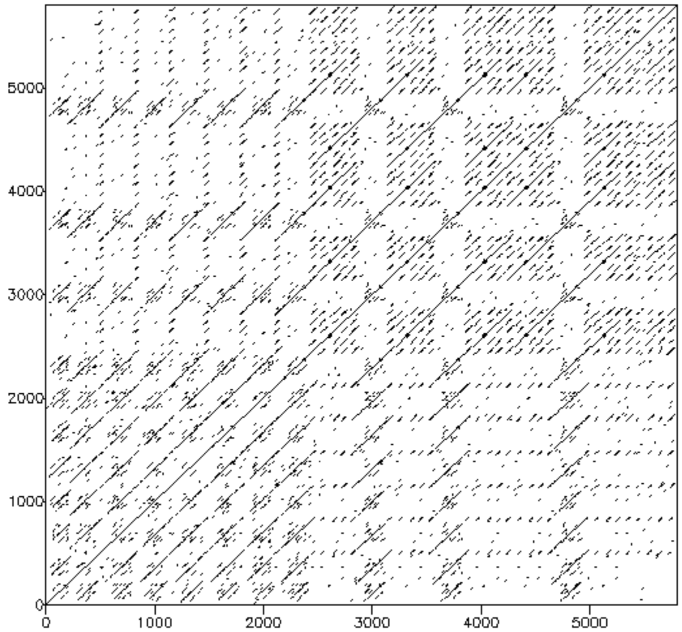

Tortoise (*Gopherus evgoodei*) EPPK1 (XP_030405357.1)

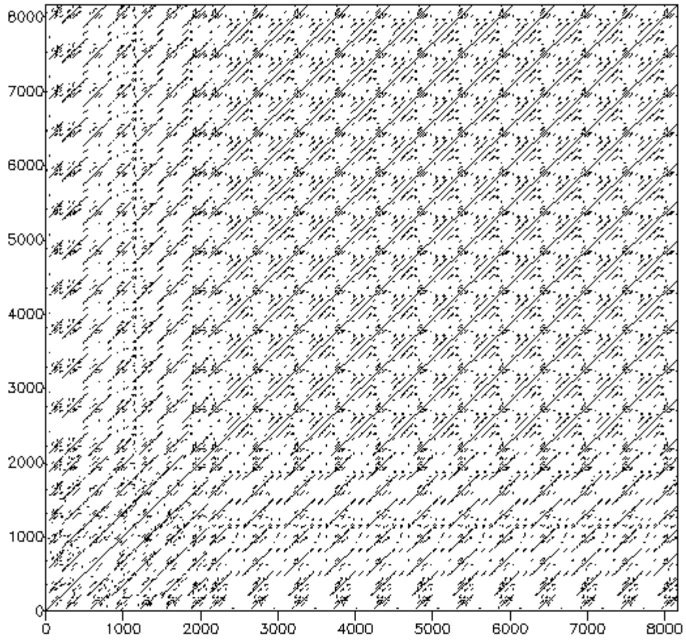

Two-lined caecilian (*Rhinatrema bivittatum*) EPPK1 (XP_029448115.1)

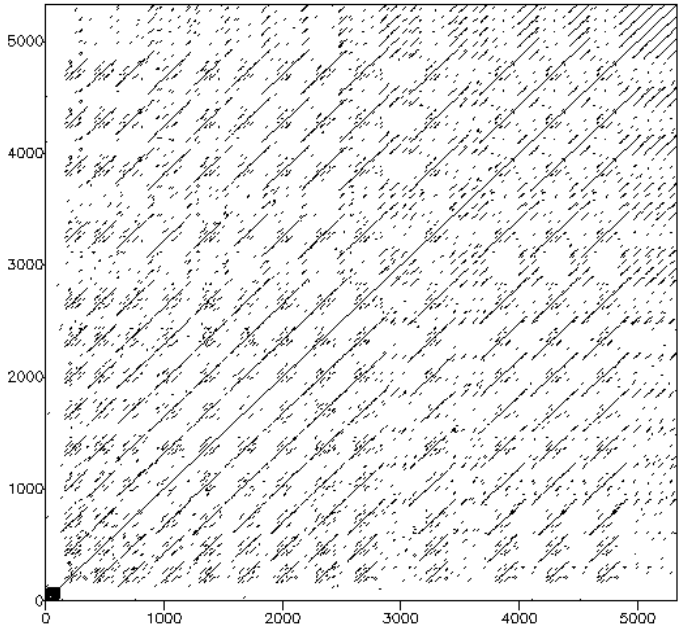

Zebrafish (*Danio rerio*) EPPK1 (NP_001313413.1)

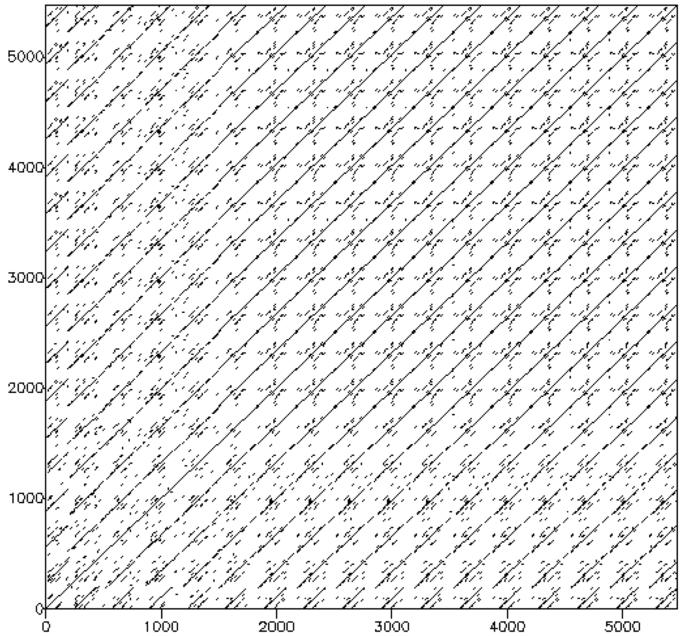

Whale shark (*Rhincodon typus*) EPPK1 (XP_020379914.1)

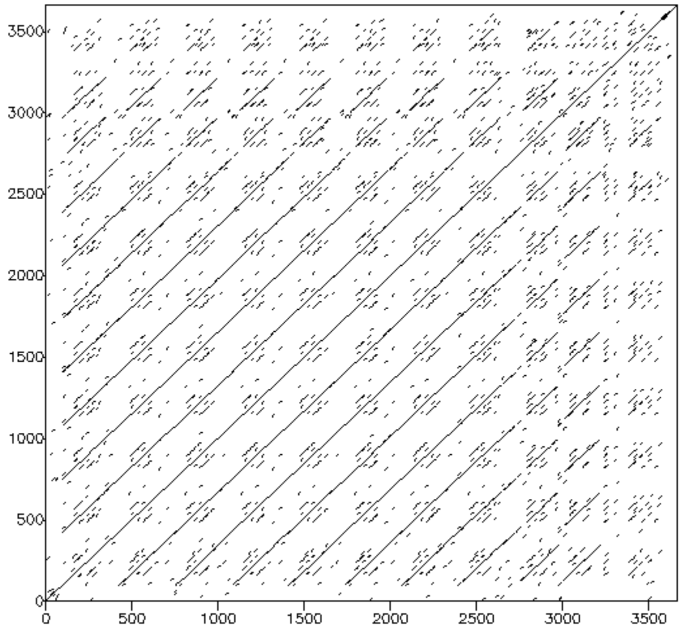

Thorny skate (*Amblyraja radiata*) EPPK1 (XP_032896528.1)

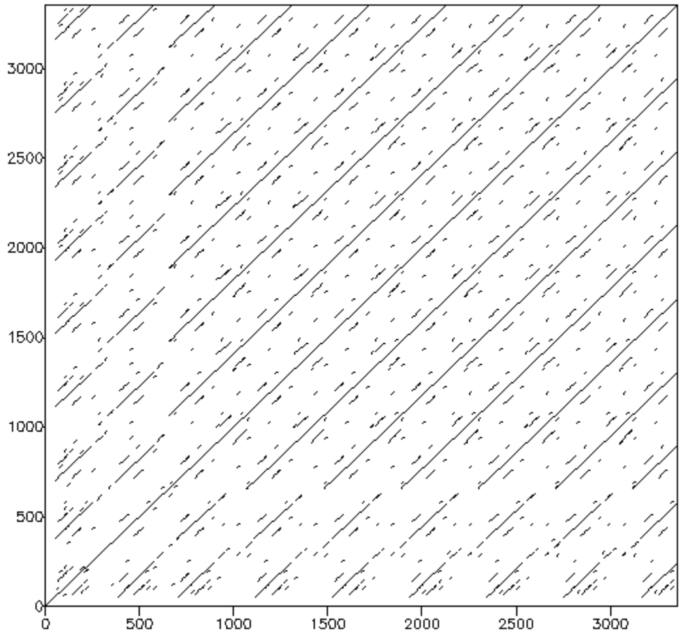

Supplementary Figure S2. Repeat structure of EPPK1 proteins. The amino acid sequences of EPPK1 proteins with the indicated GenBank accession numbers were subjected to dotplot analysis by Dotmatcher (https://www.bioinformatics.nl/cgi-bin/emboss/dotmatcher) using default settings of "Window size over which to test threshold": 10 and "Threshold": 23. Numbers on the X and Y axes indicate amino acid residues of the respective protein.

>Hippopotamus amphibius EPPK1 partial (translation of PVJP02910133.1, nucl. 435767-442636)
MNGHAAPPLDLRVTSGTETAAVPKTVKAVLGASSPSGPPAKAAASTRAGSISGVYVEASGQARSLYAAMEQGLLSSELGLALLEAQA
ATGGLVDPAQGRLLLVPEALQQGLVGLELKEKLLAAERAVTGYPDPYGGGKLALFQAIGKGVVDRALGWGWLEAQLATGGLVDPIQG
MRVAPELACQQGLLDQETWRELSELGPGSGAPGFLDPNTLERLPYRELLGRCVQAPSTGLALLPLRITFRTLSGAVSSAELLEVGVL
DEETAQGLREGRLAAPDVGTRAEVQRYLHGTGGVAGVVLLPAGHKKSFFQAAAEHLLPMGAALPLLEAQAATRTLVDPATGRQLCVD
AAVRAGLVGPELHGPLLTAEQAVTGYHDPFSGTLIPLFQAMKKELVDRPLALRLLDAQLATGGLVCPARRLRLPLEAALRFGCLDKE
TQQHLLQAAGFVDPGTQESLSYRELLARCVTDPETGLTFLPISVRSPGEEPQGPPFIEHSTQQALRAATATISVGEFQGRPQSLWEL
LFSEAVPVGQRVTLTQQHRDGALSLGELAAALRAAHEQATAAARTTFAGLRVPVTPGELLGAGIIDQDVYERLERGQTTAQAVGSLH
AVQRYLRGTGCVAGLLLPGSQEPLGVHEACRKGLLRPGTALILLEAQAATGFIIDPKENKRYSVEEALRAGVIGPDMFAKLLSAERA
VTGYTDPYTGEQISLFQAMKKDLIVRDHGIRLLEAQIATGGVIDPVHSHRVPVDVAYQRGYLDEEMSRVLEDPSDDTKGFFDPNTHE
NLTYVQLLERCVRDPDTGLYLLPLTSKRPPLVDAATRQAFQSLKLLVKHGRFRGQRVSAWALITSECVREECRRQLLQQFRRHQLSL
EQVSELLEREVERWADVTLPALRGRVTACQLREAHIIDQELLDQVLAGTVSPEALLRMDSVRSYLLGSGAVGGVLLQPSNRPLSIYQ
AMKQKLLGPGVALALLEAQAATGGMMDPHSAETMSVGEAVRRGLVGPELYGRLRRAEEAVTGFVDPFSGERVSLFQAMKKGLVPADQ
AVRLLEAQVATGGVIDPKGHHRLPVSVGTQRGCIDRDTVSALSSSETFPTPDSRGHTSYAQLLEQSVRHETSGLHLLPLPESAPAVP
TDEQVQETLKATPGAEGGTSLWELLASCHFTEEQRRGFLEDFRVGKTTVQQLQAAVRRRVQGAELLAQARLSLSGPRGRVPITWLLD
AGVISQETLEALARGKLSPAEVAVQPVVQACLWGTGCVAGVLLQPSGAKATIAQAVRDGLMPAGLGQRLLEAQVASGSLVDPLTNQR
LSVEGAVKAGLVAGALSEQLWQVERAVAGYVDPCSGGALSLWQAVQKGLVPQSEGFPLLQAQLATGGAVDPVHGVHLPQPAACRLGL
LDEQTSRVLTSTDEDSKFFFDPSTRERVTYGQLKERCVLDADTGLWLLPLPQDTALEVDDHTAVALRAMKVPVGAGRFRGLSMSLWD
LLHSEYVGLCRRRELAALCQSGRATALRQVVSAVTTLIEASERQHSQAAFRGLRKQLSARDLFRSQLIDKKTLDELTQGKKTVQEVT
EMDSVRQFLEGGNFIAGVLVQDTEEKMSISEAMWKGVLRPGTALVLLEAQAATGFIIDPVRNQKLSVEEAFAAGMFGRETYQKLLSA
ERAVTGYTDPYTGEQISLFQAMKKDLIVRDHGIRLLEAQIATGGIIDPVHSHRVPVDVAYQRGYFDAEMNRVLQDPSDDTKGFFDPN
THENLTYLQLLERCVEDPETGLYLLQVVKKGETYVYTDEATRQALQSRTTKMHVGRFADQMVSFWDLLSSPYFTEERKRGLLREYRA
QKVSLEQLLKVITTTVEDTEKQNQAIKVAGIGGEVTAAELFNSGVIDKKTLDTLHRRGAGGQDLCQLQHVKTSLEGSGCIAGVTTPS
TQEVVSVYEASRKGLIPPGFAAQLLEAQAATGFMLDPHGHQRLSVDEAVAAGLVGEELQGRLLRAEKAARGYTDPATGHTISVFEAM
KKNMLQRELGLRLLEVQVATGGIVDPLHHHRLPLETAYRRGLLDRDTHPLVAEQKCMNKRFVDPNTQEKVTYQELQQRSHREQGRDW
ALFPVISDARDPLFIDQATRKALEAERVEVTVGRFKGQRPSVWELLNSEYLTEDKKLELVLKYKTDTTHALQKVVKVIIEVIEEKEE
GNEQLWFRGIRRQVTASELLQSGIITQDMMQDLQAGRSRAEDLTESEVVKRYLEGTSCIAGVLVPAKDEPGRQEKMSIYQAMWKGVL
RPGTALVLLEAQAATGFVIDPVRNQKLS

Supplementary Figure S3. Partial amino acid sequence of hippopotamus EPPK1. *Hippopotamus amphibius* isolate BS01 ScHSfme_924630;HRSCAF=982430, whole genome shotgun sequence, GenBank accession number PVJP02910133.1 was translated in the range of nucleotides 435767-442636. The amino acid sequence of EPPK1 is incomplete at the carboxy-terminus because the sequence contig ends before the stop codon.

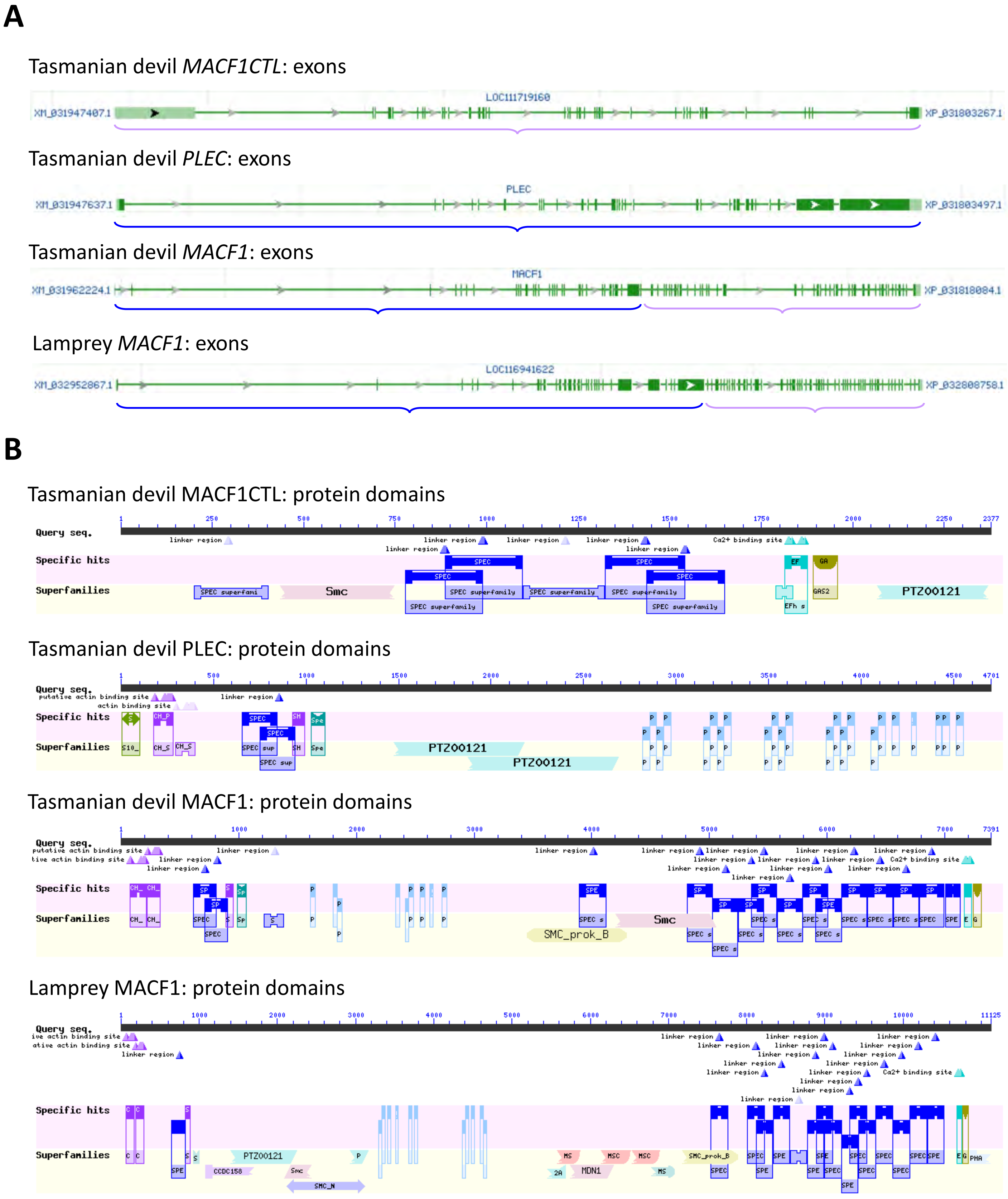

Supplementary Figure S4. Structures of MACF1, PLEC and MACF1CTL. (A) Exon-intron structures of *MACF1CTL*, *PLEC* and *MACF1* of the Tasmanian devil (*Sarcophilus harrisii*) and of *MACF1* of the lamprey (*Petromyzon marinus*). Gene information is shown in Supplementary Table S1. The graphics display exons as green boxes in the standard NCBI Gene view. Homologous gene segments are marked by colored lines under the genes. **(B)** Protein domain organization of MACF1CTL, PLEC and MACF1 of the Tasmanian devil (*Sarcophilus harrisii*) and of MACF1 of the lamprey (*Petromyzon marinus*). The graphics display protein domain in the NCBI Conserved Domains (Concise Results) view using default settings and domain symbols. Domains are identified by NCBI's CD-Search tool using protein query sequences to search the Conserved Domain Database. Detailed information about the gene organization and the encoded protein domains of *Sarcophilus harrisii MACF1CTL* and other genes is available at the NCBI website: https://www.ncbi.nlm.nih.gov/gene/?term=111719160 (gene) and https://www.ncbi.nlm.nih.gov/Structure/cdd/wrpsb.cgi?INPUT_TYPE=live&SEQUENCE=XP_031803267.1 (protein domains).
